# Resource requirements for essential universal health coverage: a modelling study based on findings from *Disease Control Priorities*, 3rd edition

**DOI:** 10.1016/S2214-109X(20)30121-2

**Published:** 2020-05-21

**Authors:** David A Watkins, Jinyuan Qi, Yoshito Kawakatsu, Sarah J Pickersgill, Susan E Horton, Dean T Jamison

**Affiliations:** a Division of General Internal Medicine, Department of Medicine, University of Washington, Seattle, WA, USA; b Department of Global Health, University of Washington, Seattle, WA, USA; c Office of Population Research, Princeton University, Princeton, NJ, USA; d School of Public Health and Health Systems, University of Waterloo, Waterloo, ON, Canada; e Institute for Global Health Sciences, University of California San Francisco, San Francisco, CA, USA

## Abstract

**Background:**

*Disease Control Priorities*, 3rd edition (DCP3), published two model health benefits packages (HBPs). This study estimates the overall costs and individual component costs of these packages in low-income countries (LICs) and lower-middle-income countries (lower-MICs).

**Methods:**

This study reports on our Disease Control Priorities Cost Model (DCP-CM), developed as part of the DCP3 project to determine the overall costs of the 218 health sector interventions recommended in the model HBP termed essential universal health coverage (EUHC). Model inputs included data on intervention unit costs, demographic and epidemiological data to quantify the populations in need of specific interventions, baseline coverage indicators, and estimates of required health system costs to support direct service delivery. The DCP-CM was informed primarily by published estimates of economic costs of interventions measured from the health system perspective. We estimated counterfactual annual costs for the year 2015. We disaggregated costs according to intervention characteristics (delivery platform, delivery timing, and health system objective) and did one-way and probabilistic sensitivity analyses with determination of 95% credible intervals (Crls).

**Findings:**

At 80% population coverage, the annual cost of EUHC would be US$79 (95% Crl 60–110) per capita (in 2016 US dollars) in LICs and US$130 (100–180) per capita in lower-MICs. As a share of 2015 gross national income (GNI), additional investments would require 8·0% (95% Crl 5·7–11·3) in LICs and 4·2% (2·9–5·9) in lower-MICs. A highest priority subpackage comprising 115 of the EUHC interventions would cost approximately half of these amounts (3·7% [2·6–5·3] of 2015 GNI in LICs and 2·0% [1·4–2·8] in lower-MICs). Mortality-reducing interventions would require around two-thirds of the overall package costs, with interventions to reduce mortality at age 5–69 years from non-communicable disease and injury comprising the highest share of total EUHC costs in both income groups (37·6% [37·2–37·9] in LICs and 43·0% [42·6–43·4] in lower-MICs). Interventions addressing chronic health conditions (requiring 45·5% [44·8–46·4] 2015 GNI for LICs and lower-MICs combined) and interventions delivered in health centres (requiring 49·8% [49·5–50·2] 2015 GNI for LICs and lower-MICs combined) would each comprise the plurality of costs.

**Interpretation:**

Implementation of EUHC would require costly investment, especially in LICs. DCP-CM is available as an online tool that can inform local HBP deliberation and support efficient investment in UHC, especially as countries pivot towards non-communicable disease and injury care.

**Funding:**

Bill & Melinda Gates Foundation, Trond Mohn Foundation, and Norwegian Agency for Development Cooperation.

## Introduction

Health planners working in low-income countries (LICs) and middle-income countries (MICs) face difficult choices and trade-offs when selecting health interventions to include in publicly financed health-care systems.^1^ The constraints on the availability of public resources for health care in these countries limits access to necessary services and shifts costs to patients and their households, inevitably resulting in financial hardship.^2^, ^3^ The global movement towards universal health coverage (UHC) has highlighted the importance of expanding health-care access, and of finance arrangements that can maximise prepayment and risk pooling and minimise financial risk.^4^, ^5^, ^6^ Most national UHC systems focus their spending on a list of high-priority health interventions, frequently referred to as a health benefits package (HBP), guaranteed to the population and, in principle, offered at little to no cost at the point of care.^7^

Benefits packages have the potential to guide investment and strengthen health systems while directly addressing the health needs of populations and being mindful of budget constraints. Previous literature has explored the process by which governments develop and implement HBPs;^7^ however, guidance is also needed on the desired contents of HBPs. The World Bank's 1993 World Development Report produced one of the first model HBPs for LICs and MICs,^8^ and proposed related public health packages, which were discussed in detail in the accompanying publication *Disease Control Priorities in Developing Countries*.^9^ Since that time, health agendas in LICs and MICs have expanded, pressuring countries to include more health interventions within their HBPs and address an increased range of health needs, including non-communicable diseases and injuries.

**Research in context**

**Evidence before this study**

Our modelling drew on systematic reviews of costing and cost-effectiveness studies in low-income countries (LICs) and middle-income countries (MICs) that our colleagues developed for *Disease Control Priorities*, 3rd edition (DCP3). We supplemented these data with the DCP3 repository of unpublished literature on health system costs and by a PubMed search for studies published in English from Jan 1, 2005 until July 19, 2019, on the cost of health benefits packages (HBPs) in LICs and MICs, using the search terms “universal health coverage” and “cost”. We identified only a handful of published large-scale costing exercises, which we compare and contrast with our analysis.

**Added value of this study**

In contrast to previous studies, which focused largely on general investments in health systems, our analysis focuses specifically on packages of evidence-informed health interventions within HBPs. We provide an alternative set of estimates of the cost of universal health coverage (UHC) that is in broad alignment with estimates from previous studies, but which has a somewhat different composition. Our analytical tool, the Disease Control Priorities Cost Model (DCP-CM), assesses the cost composition of our model in a number of important dimensions, such as costs related to delivery platforms, health system objectives, and intervention timing characteristics. These disaggregated analyses add value to global and national discussions on UHC financing and can help governments generate packages that address emerging challenges, such as non-communicable diseases and injuries, and neglected delivery platforms, such as first-level hospitals.

**Implications of all the available evidence**

Similar to other previous studies, we have found that the achievement of a defined vision for UHC would require large-scale new spending, particularly in LICs. To support these investments, a number of countries will require sustained development assistance for years to come. Additionally, demographic and epidemiological transitions will probably increase the cost of HBPs, especially in MICs, requiring greater public investment over time.

Decision makers can benefit from up-to-date information on the potential costs of an HBP to guide resource allocation and plan the expansion of priority interventions. For health ministries with highly stretched technical expertise and sparse local data, information on candidate HBP interventions and their potential costs can simplify the decision space and guide local evidence gathering and synthesis.

In this study, we estimate the potential cost of a model HBP, termed essential UHC (EUHC), that was developed in the latest edition of *Disease Control Priorities* (DCP3), and we explore the implications of the costs of EUHC for the health systems and UHC agendas in LICs and lower-MICs. Only a small number of published studies have costed a wide range of interventions similar to those recommended in DCP3, and therefore the scientific literature lacks consensus on standard approaches, methods, and data sources.^5^, ^10^, ^11^, ^12^ DCP3 was tasked with providing generic recommendations for LIC and MIC settings, rather than prescribing actions in specific countries. Consequently, our cost modelling illustrates typical costs of EUHC from a national perspective in so-called stylised LICs and lower-MIC settings. We further disaggregate costs according to how and when an intervention is delivered, and its primary health objectives, all of which have implications for policy.

## Methods

### Study design

As part of the DCP3 project, we developed a Disease Control Priorities Cost Model (DCP-CM) for the 218 health sector interventions recommended in the DCP3 model EUHC. We describe the methods used in this study in the context of the DCP3 project. The appendix provides further background on DCP3 and includes an in-depth description of study methods and data sources. Definitions of key concepts and terms used in this study are provided in the panel.

Panel **Definitions of key concepts used in this study**

**Essential intervention package**

A list of health interventions recommended as essential for low-income countries (LICs) and lower-middle-income countries (lower-MICs). *Disease Control Priorities*, 3rd edition (DCP3), included 21 essential intervention packages, organised around a health topic or professional community (eg, cancer or surgery), in its nine volumes. The appendix (pp 2–4) provides further details.

**Essential universal health coverage (EUHC)**

A model health benefits package for UHC systems in LICs and lower-MICs. The DCP3 EUHC package synthesised recommendations from its 21 essential packages into 218 unique interventions delivered on health system platforms (see later definition). Health interventions delivered through non-health sectors (eg, tobacco taxes, ambient air pollution regulations, and safe roads) were synthesised elsewhere in DCP3.^13^

**Highest priority package (HPP)**

A subpackage of EUHC that accounts for the resource limitations and health needs of LICs in particular. The HPP in DCP3 included 115 EUHC interventions that fulfilled more restrictive criteria than the other 103 interventions featured in the 21 essential intervention packages. The appendix (pp 3–4) provides further details.

**Platform**

A physical and organisational channel for delivering logistically related health interventions. DCP3 developed an illustrative typology of five distinct health system platforms: (1) population-based, (2) community, (3) health centre, (4) first-level hospital, and (5) referral and specialty hospitals (appendix p 3).

**Delivery timing characteristic**

A method of classifying health interventions according to the frequency and urgency of client–provider interactions. DCP3 classified interventions as (1) urgent, (2) chronic, or (3) time-bound (appendix p 3).

**Health system objective**

A method of classifying health interventions according to the primary outcomes they affect. In the present study, we classified interventions as primarily affecting (1) under-5 mortality, (2) mortality aged 5–69 years from communicable, maternal, perinatal, and nutritional conditions, (3) mortality aged 5–69 years from non-communicable diseases or injuries, (4) disability (from any cause), or (5) non-health outcomes (in this study, palliative care, met need for contraception, cosmetic surgery, psychosocial support, and intellectual development during childhood).

**Country income group**

DCP3 modelled costs for two groups of countries: LICs and lower-MICs. The July 2014 World Development Indicators classification of countries^14^ was used in DCP3 and thus in this analysis. In 2015, the population of LICs (as defined in this study) was 0·90 billion, and the population of lower-MICs was 2·7 billion. The appendix (pp 19–20) lists the countries in each of these groups.

**Service delivery costs**

Direct costs required to deliver a health intervention to a particular client; these include provider costs, equipment costs, and drugs and consumables.

**Health system costs**

Costs that are required to support the delivery of health interventions but are not easily allocable to specific health interventions. DCP3 split health system costs into facility-level costs (eg, pathology services, administration, and utilities and maintenance) and so-called above-facility systems costs (eg, supply chain and financing) and estimated these as functions of service delivery costs.

**Incremental cost**

In the context of this modelling study, incremental cost is the additional cost required to maintain the coverage of an intervention at a target level (eg, 80% of the population).

### Essential interventions and EUHC in DCP3

DCP3 was a 5-year international effort of over 500 collaborators that sought to identify priority areas for health interventions in LICs and. Recommendations from DCP3 were published in 172 chapters in nine volumes that went through a peer review process overseen by the US National Academies of Sciences, Engineering, and Medicine. The DCP3 volumes included a series of 21 essential intervention packages (ie, lists of health interventions) that met three criteria: (1) provided good value for money (ie, cost-effective), (2) were feasible to implement in LICs and MICs, and (3) addressed a considerable disease burden. Intervention cost-effectiveness was assessed with published economic evidence and judgment from technical experts about the transferability and generalisability of this evidence. The present study was undertaken at the end of the DCP3 project and did not inform package development. The 21 intervention packages were organised around particular health issues (eg, cancer or reproductive health) or professional communities (eg, surgery or pathology). The packages included both health sector interventions (eg, immunisation and HIV treatment) and intersectoral policies (eg, tobacco taxation and road safety; appendix pp 2–4).^9^

During the DCP3 project, Watkins and colleagues^15^ synthesised the health sector interventions from across the 21 essential packages outlined in DCP3 into a model HBP, comprising a list of 218 unique interventions termed EUHC. The rationale for developing EUHC was to provide model delineations on the contents of HBPs in LICs and lower-MICs. EUHC was designed as a starting point for local deliberation on HBP reform, not a prescription for the HBP of any particular country. A list of 115 of the 218 EUHC interventions, termed the highest priority package (HPP), was developed by the authors with a deliberative process described in the appendix (pp 3–4). HPP interventions were deemed particularly urgent to implement, especially in LICs, which face greater resource constraints than MICs.

### Costing approach

Our team generated preliminary cost estimates of EUHC that were featured in DCP3 and a report for the 2013 *Lancet* Commission on Investing in Health.^16^, ^17^ The present study describes the formal cost modelling related to this work (ie, the DCP-CM). The question we posed was: how much would EUHC cost in the current epidemiological environment, if a typical LIC or lower-MIC had already fully implemented the package? To answer this question, we estimated counterfactual costs for the year 2015 using aggregate epidemiological and demographic data for LICs and lower-MICs in the year 2015. We term our approach counterfactual because we looked at the cost implications of delivering all 218 EUHC interventions at 80% population coverage in 2015 compared with their actual coverage in 2015. 80% coverage was selected as a realistic and consistent target for interventions during the sustainable development goal (SDG) era.

The DCP-CM was informed primarily by published estimates of economic costs of interventions, measured from the health system perspective. Our approach was designed to quantify the magnitude and composition of costs of EUHC and the HPP as a generic, but also concrete and structured, starting point for national or subnational dialogues on priority setting for UHC. It was not designed to estimate year-over-year budgetary consequences of EUHC under particular scale-up scenarios in specific countries. Our estimates of annual incremental costs therefore reflect the difference between maintaining all package interventions at a counterfactual 80% coverage in 2015 and maintaining each intervention at its current coverage in 2015. Annualised long-run average costs of labour and capital replacement are included in the incremental costs.

### Data sources

We used a variety of data sources to populate DCP-CM, described in the appendix (pp 6–11) and our DCP-CM online tool. Demographic and epidemiological estimates from the literature and from international organisations were used to quantify the population in need of each intervention. We aggregated country-specific, population-in-need data into LIC and lower-MIC groups according to the World Bank classification of each country as of July, 2014,^14^ which was the classification used throughout DCP3 (appendix pp 19–20). When available, estimates of baseline intervention coverage were taken from WHO or from the literature. In many cases, coverage indicators for tracer interventions (ie, those with available data to track coverage) were used as proxies for coverage of similar interventions. For several interventions, no proxies were available, and thus baseline coverage was generated on the basis of expert opinion or assumption. Coverage data for each intervention is given in our DCP-CM tool.

Unit costs for interventions were usually taken from the literature with a selection process described in the appendix (pp 7–8). Consistent with the literature, we define the unit cost as the total cost of delivering the intervention to one beneficiary during a standardised time period, such as patient-year for chronic interventions and per episode for acute interventions.^18^ The exceptions were a few intervention areas in which DCP3 authors had done original cost analyses, including palliative care, rehabilitation, and some interventions in the musculoskeletal disorders and congenital and genetic disorders packages.^15^, ^19^ We used estimates of annualised long-run average costs, meaning that whenever possible we used studies that appropriately accounted for depreciation of capital. We used long-run average costs instead of marginal costs, because the modelled expansion of intervention scope and coverage was typically large compared with baseline values, and large fixed and capital investments would be needed to implement EUHC. We were not, however, able to adjust for differential discount rates without access to primary data for many studies.

### Perspective of the cost analysis

All unit cost estimates were converted and inflated to 2016 US dollars (appendix p 8). We used exchange-rate US dollars rather than US dollars adjusted for purchasing power parity because we sought to estimate costs from a supposed national perspective for our two stylised countries (LICs and lower-MICs). We thus valued the cost of internationally tradeable inputs at prices determined by the market exchange rate, and the cost of goods and services at country-specific prices that reflect health worker salary differences (which in turn vary with per-capita gross national income [GNI]).

Service delivery cost estimates from studies of specific countries were adjusted to mean LIC and lower-MIC costs with ratios of per-capita GNI for non-traded inputs and assumptions about the proportion of the unit cost attributable to traded goods (appendix pp 8–9). The service delivery costs were further marked up for additional health system costs, which included both facility-level costs (eg, rent, utilities, and maintenance) and so-called above-facility costs (eg, health financing, supply chains and logistics, health information systems, and administration). Our definition of above-facility costs was aligned with a 2009 working group report of the WHO High Level Taskforce on Innovative International Financing for Health Systems (appendix pp 10–11).^10^ We assumed that the mark-ups used for facility-level and above-facility costs included in-service training costs.

### Other modelling considerations

We did one-way and probabilistic sensitivity analyses on key parameters in our cost model (appendix pp 11–12). Ranges of plausible values were propagated throughout our model in the probabilistic uncertainty analysis that used 10 000 Monte Carlo simulations to generate 95% credible intervals (95% CrIs) for total and incremental costs. In this analysis we looked at the distribution of EUHC and HPP costs across three different types of intervention characteristic: delivery platform, delivery timing, and health system objective. The panel defines these terms and lists the categories used for each characteristic. Parameters for the simulations were assumed to be independently distributed, as no information was available on joint distributions.

The online tool for the DCP-CM that we created includes all data, all data sources, and assumptions used in this analysis. This tool allows the user to edit default inputs (and recalculate HBP costs) and download data inputs and outputs. For example, if a user were concerned that we had underestimated labour costs, they could simply replace our unit cost estimates with their own. Additionally, if a particular health condition (eg, malaria) was not a concern of the user, interventions addressing that condition could simply be deselected from the package and excluded from the costs.

### Role of the funding source

The funders of the study had no role in study design, data collection, data analysis, data interpretation, or writing of the report. The corresponding author had full access to all the data in the study and had final responsibility for the decision to submit for publication.

## Results

As inputs to the cost of EUHC, we estimated the annual costs of the 21 essential intervention packages in DCP3 (table 1). In both LICs and lower-MICs, only a few packages accounted for much of the total costs. The packages each comprising 10% or more of overall EUHC costs were for cardiovascular, respiratory, and related disorders (29% in LICs and 36% in lower-MICs) and for HIV and sexually transmitted infections (12% in LICs).

**Table 1:** Annual costs of the 21 essential intervention packages in DCP3

		**Baseline cost per capita**	**Baseline cost for overall population, billions US$**	**Incremental cost per capita**	**Incremental cost for overall population, billions US$**	**Total cost per capita**	**Total cost for overall population, billions US$**	**Package share of total costs**
**Low-income countries**								
Age-related								
	1. Maternal and newborn health (volume 2)	$1·1	$1·0	$1·6	$1·4	$2·7	$2·4	5·0%
	2. Child health (volume 2)	$1·9	$1·7	$0·92	$0·82	$2·8	$2·5	5·3%
	3. School-age health and development (volume 8)	$0·12	$0·11	$0·24	$0·21	$0·36	$0·32	0·7%
	4. Adolescent health and development (volume 8)	$0·39	$0·35	$0·55	$0·50	$0·94	$0·85	1·8%
	5. Reproductive health and contraception (volumes 1, 2, and 8)	$0·77	$0·69	$0·40	$0·36	$1·2	$1·0	2·2%
Infectious diseases								
	6. HIV and STIs (volume 6)	$2·3	$2·0	$4·0	$3·6	$6·3	$5·7	11·9%
	7. Tuberculosis (volume 6)	$0·39	$0·36	$0·17	$0·15	$0·56	$0·51	1·1%
	8. Malaria and adult febrile illness (volumes 2, 6, and 8)	$1·5	$1·4	$2·4	$2·2	$0·39	$3·5	7·5%
	9. Neglected tropical diseases (volume 6)	$0·22	$0·20	$0·62	$0·56	$0·84	$0·75	1·6%
	10. Pandemic and emergency preparedness (volume 9)	$0·015	$0·014	$0·68	$0·61	$0·72	$0·65	1·4%
Non-communicable disease and injury								
	11. Cardiovascular, respiratory, and related disorders (volume 5)	$0·85	$0·76	$15	$13	$15	$14	29·2%
	12. Cancer (volume 3)	$0·16	$0·14	$2·1	$1·9	$2·3	$2·1	4·3%
	13. Mental, neurological, and substance use disorders (volume 4)	$0·23	$0·21	$2·5	$2·2	$2·7	$2·5	5·2%
	14. Musculoskeletal disorders (volume 9)	$0·060	$0·054	$1·6	$1·5	$1·7	$1·5	3·2%
	15. Congenital and genetic disorders (volume 9)	$0·45	$0·40	$1·0	$0·94	$1·5	$1·3	2·8%
	16. Injury prevention (volume 7)	$0·0021	$0·0019	$0·049	$0·044	$0·051	$0·046	0·1%
	17. Environmental improvement (volume 7)	$0·044	$0·040	$0·050	$0·045	$0·094	$0·084	0·2%
Health services								
	18. Surgery (volume 1)	$0·27	$0·24	$4·8	$4·4	$5·1	$4·6	9·7%
	19. Rehabilitation (volume 9)	$0·12	$0·11	$1·9	$1·7	$2·0	$1·8	3·7%
	20. Palliative care and pain control (volume 9)	$0·11	$0·10	$1·6	$1·5	$1·7	$1·6	3·3%
	21. Pathology (volume 9)	$0·47	$0·42	$2·2	$2·0	$2·7	$2·5	5·2%
Totals								
	Total service delivery costs	$11	$10	$42	$38	$53	$47	..
	De-duplicated service delivery costs	$8·8	$7·9	$36	$33	$45	$41	57·2%
	Total health system costs	$6·6	$6·0	$28	$25	$34	$31	42·8%
	Total cost (sum of service delivery and health system costs)	$15	$14	$64	$57	$79	$71	100%
**Lower-middle-income countries**								
Age-related								
	1. Maternal and newborn health (volume 2)	$1·6	$4·2	$2·1	$5·6	$3·7	$9·8	4·3%
	2. Child health (volume 2)	$2·8	$7·6	$0·97	$2·6	$3·8	$10	4·3%
	3. School-age health and development (volume 8)	$0·11	$0·29	$0·25	$0·67	$0·36	$0·96	0·4%
	4. Adolescent health and development (volume 8)	$0·49	$1·3	$0·70	$1·9	$1·2	$3·2	1·4%
	5. Reproductive health and contraception (volumes 1, 2, and 8)	$1·8	$4·9	$0·50	$1·3	$2·3	$6·3	2·8%
Infectious diseases								
	6. HIV and STIs (volume 6)	$1·6	$4·4	$5·9	$16	$7·6	$20	8·9%
	7. Tuberculosis (volume 6)	$0·43	$1·1	$0·25	$0·66	$0·68	$1·8	0·8%
	8. Malaria and adult febrile illness (volumes 2, 6, and 8)	$4·0	$11	$2·2	$5·8	$6·1	$16	7·3%
	9. Neglected tropical diseases (volume 6)	$0·32	$0·87	$0·67	$1·8	$1·0	$2·7	1·2%
	10. Pandemic and emergency preparedness (volume 9)	$0·072	$0·19	$0·65	$1·7	$0·72	$1·9	0·9%
Non-communicable disease and injury								
	11. Cardiovascular, respiratory, and related disorders (volume 5)	$9·6	$26	$21	$55	$30	$81	36·2%
	12. Cancer (volume 3)	$0·21	$0·57	$1·3	$3·6	$1·5	$4·1	1·8%
	13. Mental, neurological, and substance use disorders (volume 4)	$0·59	$1·6	$6·3	$17	$6·8	$18	8·1%
	14. Musculoskeletal disorders (volume 9)	$0·28	$0·75	$3·4	$9·1	$3·6	$9·7	4·3%
	15. Congenital and genetic disorders (volume 9)	$0·48	$1·3	$1·5	$4·1	$2·0	$5·4	2·4%
	16. Injury prevention (volume 7)	$0·0053	$0·014	$0·16	$0·43	$0·17	$0·44	0·2%
	17. Environmental improvement (volume 7)	$0·012	$0·33	$0·11	$0·30	$0·18	$0·49	0·2%
Health services								
	18. Surgery (volume 1)	$0·87	$2·3	$6·5	$17	$7·4	$20	8·7%
	19. Rehabilitation (volume 9)	$0·43	$1·1	$3·9	$10	$4·3	$11	5·1%
	20. Palliative care and pain control (volume 9)	$0·057	$0·15	$0·51	$1·4	$0·57	$1·5	0·7%
	21. Pathology (volume 9)	$0·80	$2·1	$3·1	$8·4	$4·5	$12	5·3%
Totals								
	Total service delivery costs	$26	$69	$59	$160	$85	$230	..
	De-duplicated service delivery costs	$23	$61	$53	$140	$75	$200	57·2%
	Total health system costs	$17	$46	$40	$110	$57	$150	42·8%
	Total cost (sum of service delivery and health system costs)	$40	$110	$92	$250	$130	$350	100%

All costs are in 2016 US dollars rounded to two significant figures. Baseline costs reflect intervention costs at current coverage. Incremental costs are those required to increase coverage of all interventions from baseline to 80%. Total costs are the sum of baseline costs and incremental costs and reflect the cost of sustaining all interventions at 80% coverage. The baseline cost estimate does not differentiate between public and private sources. The DCP3 volumes in which the essential packages are featured are indicated. Total costs in the final row are the sum of de-duplicated service delivery costs and total health system costs. The de-duplicated service delivery costs are substantially lower than the total service delivery costs because a number of interventions are (intentionally) included in more than one DCP3 essential package. The shares of costs presented for each of the 21 essential packages use the de-duplicated service delivery costs as the denominator; therefore the sum of these shares exceeds 100% because of duplication. However, the share of any given package can be interpreted as the remaining fraction of the total EUHC service delivery cost if the interventions in all other packages were removed. The shares of costs presented in the totals sections reflect the relative proportion of EUHC costs related to service delivery and to health system strengthening, with the sum of these two being the total cost of EUHC. DCP3=*Disease Control Priorities*, 3rd edition. STI=sexually transmitted infection. EUHC=essential universal health coverage.

In LICs, the total annual cost of EUHC at 80% coverage would be US$71 billion (95% Crl 54–95), or US$79 (60–110) per capita (table 2). The incremental annual cost of reaching 80% coverage of all EUHC interventions would be US$57 billion (41–80), or US$64 (46–90) per capita. In lower-MICs, the total annual cost of EUHC would be US$350 billion (270–470), or US$130 (100–180) per capita, and the incremental annual cost would be US$250 billion (170–350), or US$92 (65–130) per capita. The incremental annual cost of the EUHC package would comprise 8·0% (95% Crl 5·7–11·3) of 2015 GNI in LICs and 4·2% (2·9–5·9) in lower-MICs. The HPP subset, comprising 115 of the EUHC interventions, would be roughly half of the cost of the EUHC package in each of the country income groups (table 2). The incremental annual cost of the HPP would comprise 3·7% (2·6–5·3) of 2015 GNI in LICs and 2·0% (1·4–2·8) in lower-MICs.

**Table 2:** Total and incremental costs of the DCP3 model health benefits packages in LICs and lower-MICs

	LICs*		Lower-MICs*	
	HPP	EUHC	HPP	EUHC
Incremental annual cost*, billions US$	$27 (19–38)	$57 (41–80)	$120 (80–170)	$250 (170–350)
Incremental annual cost per capita	$30 (21–42)	$64 (46–90)	$43 (30–62)	$92 (65–130)
Total annual cost*, billions US$	$36 (27–48)	$71 (54–95)	$180 (140–240)	$350 (270–470)
Total annual cost per capita	$40 (30–54)	$79 (60–110)	$69 (52–92)	$130 (100–180)
Incremental annual cost as a share of 2015 GNI per capita	3·7% (2·6–5·3)	8·0% (5·7–11·3)	2·0% (1·4–2·8)	4·2% (2·9–5·9)
Total annual cost as a share of 2015 GNI per capita	5·1% (3·8–6·8)	10·0% (7·5–13·3)	3·1% (2·3–4·1)	6·0% (4·5–8·0)

All costs are in 2016 US dollars rounded to two significant figures. In 2015, LICs had a population of 0·90 billion and a GNI of US$0·70 trillion, and lower-MICs had a population of 2·7 billion and a GNI of US$5·9 trillion. Across all LICs and lower-MICs, the incremental annual cost of EUHC would be US$310 billion (population-weighted mean $85 per capita), and the total annual cost of EUHC would be US$420 billion (population-weighted mean $120 per capita). Values in parenthesis represent the 95% credible intervals computed in the probabilistic sensitivity analysis. DCP3=*Disease Control Priorities*, 3rd edition. LIC=low-income country. Lower-MIC=lower-middle-income country. HPP=highest priority package. EUHC=essential universal health coverage. GNI=gross national income.

* Incremental and total cost estimates reflect a target of 80% population coverage.

The figure illustrates the distribution of incremental costs according to intervention delivery platform and delivery timing characteristics for LICs and lower-MICs combined, with individual results being similar between each country income group (data not shown). The share of EUHC costs by delivery platform were 1·6% (1·5–1·6) for population-based health interventions, 11·8% (11·0–12·5) for community, 49·8% (49·4–50·2) for health centre, 31·0% (30·6–31·5) for first-level hospital, and 5·8% (5·4–6·1) for referral and specialty hospitals. The share of EUHC costs by delivery timing characteristics were 28·5% (27·6–29·1) for urgent interventions, 45·5% (44·8–46·4) for chronic interventions, and 26·0% (25·5–26·4) for time-bound (non-urgent) interventions.

**Figure:**
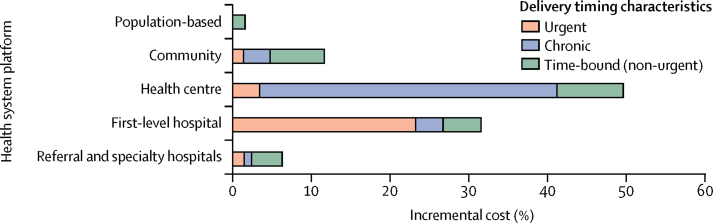
Distribution of incremental costs of essential universal health coverage according to intervention characteristics The five stylised health system delivery platforms have been described in detail by Watkins and colleagues.^15^ All components across the five platforms and the three types of delivery timing sum to 100%. Shares are for the incremental costs of low-income countries and lower-middle-income countries combined into one graph, as individual results were similar for each of the two country income groups.

EUHC and HPP costs were also analysed according to their principal health system objective (table 3, appendix p 14). We specifically included non-health outcomes as an objective because health systems typically deliver a range of interventions with a primary objective other than to reduce mortality or disability (eg, contraception and palliative care). Interventions to reduce under-5 mortality comprised the highest share of total HPP costs (28·0% [27·5–28·6]) in LICs, whereas interventions to reduce mortality at age 5–69 years from non-communicable disease and injury comprised the highest share of total HPP costs (33·6% [33·3–33·9]) in lower-MICs, and of total EUHC costs (37·6% [37·2–37·9] in LICs and 43·0% [42·6–43·4] in lower-MICs) in both income groups. In general, 19–25% of the cost of each package in either income group would be directed towards disability reduction rather than mortality. Interventions addressing non-health outcomes comprised a lower share of EUHC costs than HPP costs, and a lower share of costs in lower-MICs than LICs. Interventions to reduce mortality at age 5–69 years from non-communicable disease and injury comprising the highest share of total EUHC costs in both income groups.

**Table 3:** Distribution of model package costs by health system objective

			**Total**	Mortality reduction*: age ≤5 years	Mortality reduction*: age 5–69 years, communicable, maternal, perinatal, and nutritional conditions	Mortality reduction*: age 5–69 years, non-communicable diseases and injuries	Reduction*in disability	**Non-health outcomes**†
**Highest priority package**								
Number of interventions			115	38	18	27	15	12
	LICs							
		Total cost, % of GNI	5·1%	1·4%	0·8%	1·3%	1·0%	0·6%
		Total cost, billions US$ per year	$36	$10	$5·6	$9·0	$7·0	$4·5
		Share of overall costs	100%	28·8%	15·4%	24·9%	19·3%	12·4%
	Lower-MICs							
		Total cost, % of GNI	3·1%	0·7%	0·4%	1·0%	0·7%	0·2%
		Total cost, billions US$ per year	$180	$43	$21	$62	$44	$14
		Share of overall costs	100%	23·6%	11·2%	33·6%	23·8%	7·8%
**Essential universal health coverage package**								
		Number of interventions	218	52	44	45	58	19
	LICs							
		Total cost, % of GNI	10·0%	1·6%	1·6%	3·7%	2·2%	0·8%
		Total cost, billions US$ per year	$71	$12	$11	$27	$16	$5·6
		Share of overall costs	100%	16·2%	16·1%	37·6%	22·3%	7·8%
	Lower-MICs							
		Total cost, % of GNI	6·0%	0·9%	0·8%	2·6%	1·5%	0·3%
		Total cost, billions US$ per year	$350	$51	$44	$150	$89	$17
		Share of overall costs	100%	14·4%	12·5%	43·0%	25·1%	4·9%

All costs are in 2016 US dollars rounded to two significant figures. The elements of the essential pathology package are included in total costs but do not fit into any one objective; therefore, the numbers in the total column are slightly higher than the sum of the five objectives. For presentational purposes, 95% credible intervals from the probabilistic sensitivity analysis are not shown but are provided in the appendix (p 14). LIC=low-income country. GNI=gross national income. MIC=middle-income country.

* Any amount of reduction.

† As listed in the panel.

Our one-way sensitivity analysis showed that the incremental costs were most sensitive to changes in intervention unit cost and baseline coverage, and less sensitive to changes in facility-level health system costs, above-facility health system cost, uncertainty in population-in-need estimates, and uncertainty in total fertility rate estimates (appendix p 12). Regarding results from the probabilistic sensitivity analysis (table 2), the wide range of uncertainty in several key model parameters, including unit costs (appendix p 11), led to wide 95% Crls, with the upper (97·5th) percentile value usually being around twice the value of the lower (2·5th) percentile.

## Discussion

Despite considerable growth in recent years in the literature on financing UHC, only a few studies have attempted to estimate the cost of moving toward UHC in LICs and MICs. The World Bank, in its 1993 World Development Report on investing in health, provided estimates for public health and clinical packages comprising a small number of interventions,^8^ and the WHO Commission on Macroeconomics and Health in 2002 provided estimates for a broader set of interventions focused on the targets of the Millennium Development Goals.^20^ McIntyre and colleagues,^21^ drawing on cost estimates from the WHO Commission, estimated that the minimum required expenditure on UHC in LICs would be US$86 per capita (in 2012 US dollars) annually, although they argued that 5% of gross domestic product would be a more appropriate target in countries with higher income levels. Our study can be viewed as the latest in this series of costing exercises that view HBPs (and UHC) as the sum of priority interventions. In addition, we provide breakdowns of costs according to key dimensions that affect policy. Studies in recent years by Stenberg and colleagues^11^ at WHO and by Moses and colleagues^22^ at the US Institute for Health Metrics and Evaluation have also looked at the cost of achieving UHC, but with less emphasis on interventions and more on health system targets and standards (table 4). A detailed comparison between the present study and the WHO study is provided in the appendix (pp 17–20).

**Table 4:** Comparison of universal health coverage costing studies since 2015

	**Stenberg et al, 2017**^11^	**Moses et al, 2019**^22^	**Current study, 2020**
Definition of UHC	Achievement of normative levels of health workforce and facility density and high coverage (eg, 95% in the most ambitious scale-up scenario) of 187 health interventions	Achievement of target rates of utilisation of inpatient and outpatient services, relative to country disease burden	Achievement of 80% coverage of 218 essential health interventions in a model HBP
Countries included in analysis	67 LICs and MICs representing 95% of the total population of these country income groups	188 countries for which utilisation rates and health spending were modelled as part of the Global Burden of Disease Study 2016	83 LICs and lower-MICs (total population of these two income groups; costs estimated separately for each income group)
Costing method	Microcosting (ingredients approach for all health interventions and related programmes)	Gross costing (ie, average expenditure per outpatient or inpatient visit as calculated from NHA data	Mixed: unit costs for direct service delivery taken from microcosting studies, with health system costs added from gross costing studies
Included costs	Direct service delivery costs of interventions and related health system strengthening costs, including costs of reaching target workforce and facility density	All components of care measured in outpatient and inpatient expenditure per NHA data (including ancillary services); frontier analysis used to identify most efficient spending per visit	Direct service delivery costs of interventions and related health system strengthening costs
Time horizon; currency-year	Scale-up of services from current levels to target levels over 2016–30; 2014 US dollars	Counterfactual estimate for 2016 applying unit cost data (from frontier analysis) to all countries; 2017 international dollars	Counterfactual estimate for 2015 (cost of 80% coverage *vs* actual coverage); 2016 US dollars
Main findings	In the scenario with greatest progress towards UHC, an additional US$370 billion annually would be required across all LICs and MICs, or population-weighted mean total health-care spending of US$270 per capita	161 countries would require a total of Int$580 billion to Int$1·2 trillion (depending on choice of country standard) to meet target utilisation rates	An additional US$310 billion annually would be required across LICs and lower-MICs, or population-weighted mean total cost of US$120 per capita

UHC=universal health coverage. HBP=health benefits package. LIC=low-income country. Lower-MIC=lower-middle-income country. NHA=US National Health Accounts.

In the context of previous research, our analysis presents a unique perspective on the cost of UHC. DCP-CM defined two packages of health sector interventions with high value-for-money (EUHC and HPP) that could facilitate the achievement of the quantitative SDG3 targets (such as reduction in neonatal or non-communicable disease mortality) and serve as a starting point for countries seeking to progressively achieve UHC targets.^23^ Our perspective on UHC thus builds on the recommendations of the *Lancet* Commission on Investing in Health^5^ and on WHO guidance on priority setting for UHC.^24^

Despite the high costs of EUHC and the HPP, these packages appear to provide reasonably good value for money. The costs reported here can be combined with previously published estimates of premature deaths that could be averted by EUHC or the HPP, to provide approximate estimates of the cost per premature death averted (appendix p 16).^9^, ^23^ For example, the EUHC interventions aimed at reducing under-5 mortality could have a cost per child death averted in LICs of US$7500.

However, reaching 80% coverage of EUHC or HPP interventions would require substantial new investments, especially in LICs. At current growth rates, public spending on health would only be US$14–18 per capita in LICs and US$47–58 per capita in lower-MICs by 2030,^17^ which is markedly less than the total annual costs of the HPP and especially of EUHC (table 2). Stenberg and colleagues also estimated large financing gaps in UHC in LICs and modest gaps in lower-MICs.^11^ Taken together, these data imply that continued official development assistance for health will be required to finance progress towards UHC in LICs for many years to come. In MICs transitioning away from development assistance, public spending on HBPs will need to grow even faster than the current rate of growth, to meet increasing public demand for interventions that address non-communicable diseases. Nevertheless, the resources required in MICs experiencing average economic growth lie well within the bounds of economic feasibility. Whether to make high-payoff investments in health is a national choice, not determined by external constraints.

Our study points to some of the costs that health systems in LICs and lower-MICs will need to consider as their health agendas broaden and demographic and epidemiological transitions accelerate. For one, the shift from the more modest HPP to the EUHC package would imply increased attention on health conditions affecting adults, particularly fatal and non-fatal non-communicable diseases and injuries. The DCP-CM includes costs for interventions and packages that address issues such as musculoskeletal disorders, rehabilitation, pandemic preparedness, and others that do not currently receive much attention in the global UHC conversation (or among funders of disease-specific initiatives) but are nonetheless the types of intervention that countries (especially MICs) might consider in their HBPs. In view of the large share of EUHC costs related to non-communicable disease and injury care, in particular in lower-MICs, intersectoral policies, such as tobacco control and road safety measures, should be implemented in parallel with an HBP, to reduce risk exposure and future disease burden and increase HBP affordability in the long term.^13^, ^25^

We also calculated the gap between current investment in packages, platforms, types of interventions, and the investments that would be needed to implement EUHC (appendix pp 14–15). Our estimates suggest that a shift in focus to expanding primary health-care facilities (health centres and first-level hospitals) and capacity will be required to address urgent and chronic health needs. In the past, donors have often preferred community-based and population-based interventions, and many national governments are currently overinvesting in referral and tertiary services.^25^ Shifting public sector spending towards primary health-care interventions, which form the backbone of EUHC, requires not only financial resources but strong political and logistical commitments. Achieving UHC is not merely a financial, technical, or rhetorical issue; successful national initiatives to provide genuine UHC will require strong social movements and political leadership, among other factors.^4^

Although the DCP-CM tool is a simple application, it is intended to be easy to understand, transparent in its assumptions, and readily modifiable, allowing the user to quickly understand and visualise the whole situation with regard to the magnitude of HBP costs, and their distribution across health system platforms and types of interventions. Nevertheless, our analysis has several important caveats and limitations. It relied on the DCP3 recommendations for health sector interventions and focused on estimating their costs, including related health system costs. In reality, health systems have a number of important functions, and many LICs and MICs are already delivering interventions not included in the DCP3 model list, and therefore the costs we have presented would be a subset of health-care costs in a given country (or will provide the opportunity to reassess whether other unmodelled interventions are high priority). Furthermore, although intersectoral health interventions are an important complement to EUHC interventions, we did not assess their costs in this study. Some intersectoral interventions, such as publicly financed water and sanitation programmes and road safety measures, have substantial social costs and costs to governments, whereas others, such as tobacco and alcohol taxes and removal of fossil fuel subsidies, are revenue-generating for governments and neutral in terms of social cost.^13^

Future costs of a package such as EUHC will differ from the costs we have presented. The pace of the epidemiological and demographic transition, which interventions a country chooses to implement first, and how quickly interventions can be scaled are all factors that would need to be considered in strategic and financial planning exercises, but these issues were out of scope for our analysis. DCP-CM was not designed to incorporate local health system arrangements, and it does not function as a budgeting and planning tool for specific countries, nor does it disaggregate costs into those that might originate from development assistance agencies as compared with national governments. Furthermore, many countries could realise considerable economies of scope or scale, not modelled here because of the absence of empirical data and country heterogeneity, and thus would have lower unit costs than we estimated. Because of data availability limitations, we relied on expert opinion or assumption for population-in-need and coverage parameters for a number of interventions. Our sensitivity analyses reinforce the need for standardised tools to collect local data of improved quality, particularly on the cost of high-priority interventions and their current coverage levels. A new costing resource is the Global Health Cost Consortium reference case, which guides the conduct of cost analyses in LIC and MIC settings.^18^ Efforts to improve the volume and quality of cost data specific to country, platform, and intervention should underpin local decision making and tools like DCP-CM.

In conclusion, the present study adds to the body of research assessing the costs of interventions and HBPs within UHC systems. Notably, we provide cost estimates to inform choices among alternative intervention disease targets, different platforms for delivering interventions, urgent care versus chronic care needs, and health system objectives. The DCP-CM tool will add value to global and national dialogue on priority investments for UHC, by providing policy makers with cost information relevant to decisions in several dimensions important to health planning**.**

## References

[1] Glassman A, Giedion U, Smith PC, Glassman A, Giedion U, Smith PC (2017). The health benefits package: bringing universal health coverage from rhetoric to reality. What's in, what's out? Designing benefits for universal health coverage.

[2] WHO (2010). World health report 2010. Health systems financing: the path to universal coverage.

[3] Xu K, Evans DB, Kawabata K, Zeramdini R, Klavus J, Murray CJ (2003). Household catastrophic health expenditure: a multicountry analysis. Lancet.

[4] Reich MR, Harris J, Ikegami N (2016). Moving towards universal health coverage: lessons from 11 country studies. Lancet.

[5] Jamison DT, Summers LH, Alleyne G (2013). Global health 2035: a world converging within a generation. Lancet.

[6] Xu K, Evans DB, Carrin G, Aguilar-Rivera AM, Musgrove P, Evans T (2007). Protecting households from catastrophic health spending. Health Aff (Millwood).

[7] Glassman A, Giedion U, Sakuma Y, Smith PC (2016). Defining a health benefits package: what are the necessary processes?. Health Syst Reform.

[8] World Bank (1993). World development report 1993: investing in health.

[9] Jamison DT, Alwan A, Mock CN (2018). Universal health coverage and intersectoral action for health: key messages from *Disease Control Priorities*, 3rd edition. Lancet.

[10] Taskforce on Innovative International Financing for Health Systems (2009). Working Group 1 report. Constraints to scaling up and costs.

[11] Stenberg K, Hanssen O, Edejer TT (2017). Financing transformative health systems towards achievement of the health Sustainable Development Goals: a model for projected resource needs in 67 low-income and middle-income countries. Lancet Glob Health.

[12] Cashin C, Özaltın A, Glassman A, Giedion U, Smith PC (2017). At what price? Costing the health benefits package. What's in, what's out? Designing benefits for universal health coverage.

[13] Watkins DA, Nugent R, Saxenian H, Jamison DT, Gelband H, Horton S (2017). Intersectoral policy priorities for health. *Disease Control Priorities*, 3rd edn: vol 9. Improving health and reducing poverty.

[14] World Bank (2014). World development indicators 2014. https://openknowledge.worldbank.org/bitstream/handle/10986/18237/9781464801631.pdf.

[15] Watkins DA, Jamison DT, Mills A, Jamison DT, Gelband H, Horton S (2017). Universal health coverage and essential packages of care. *Disease Control Priorities*, 3rd edn: vol 9. Improving health and reducing poverty.

[16] Watkins DA, Qi J, Horton SE, Jamison DT (Nov 13, 2017). Costing universal health coverage: the DCP3 model. DCP3 working paper #20. http://dcp-3.org/resources/costs-and-affordability-essential-universal-health-coverage-low-and-middle-income.

[17] Watkins DA, Qi J, Saxenian H, Horton SE (Oct 18, 2018). Costing universal health coverage: an update of the DCP3 costing model for the *Lancet* Commission on Investing in Health. DCP3 working paper #24. http://dcp-3.org/resources/costing-universal-health-coverage-update-dcp3-costing-model-lancet-commission-investing.

[18] Vassall A, Sweeney S, Kahn JG (Sept 12, 2017). Reference case for estimating the costs of global health services and interventions. https://www.ghcosting.org/pages/standards/reference_case.

[19] Krakauer E, Kwete X, Verguet S, Jamison DT, Gelband H, Horton S (2017). Palliative care and pain control. *Disease Control Priorities*, 3rd edn: vol 9. Improving health and reducing poverty.

[20] Jha P, Mills A, Hanson K (2002). Improving the health of the global poor. Science.

[21] McIntyre D, Meheus F, Røttingen JA (2017). What level of domestic government health expenditure should we aspire to for universal health coverage?. Health Econ Policy Law.

[22] Moses MW, Pedroza P, Baral R (2019). Funding and services needed to achieve universal health coverage: applications of global, regional, and national estimates of utilisation of outpatient visits and inpatient admissions from 1990 to 2016, and unit costs from 1995 to 2016. Lancet Public Health.

[23] Watkins DA, Norheim OF, Jha P, Jamison DT (Nov 13, 2017). Reducing mortality within UHC: the DCP3 model. DCP3 working paper #21. http://dcp-3.org/resources/mortality-impact-achieving-essential-universal-health-coverage-low-and-middle-income.

[24] WHO (2014). Making fair choices on the path to universal health coverage. Final report of the WHO Consultative Group on Equity and Universal Health Coverage.

[25] Watkins DA, Yamey G, Schäferhoff M (2018). Alma-Ata at 40 years: reflections from the *Lancet* Commission on Investing in Health. Lancet.

