# Supplementary material for: Resource requirements for essential universal health coverage: a modelling study based on findings from Disease Control Priorities, 3rd edition
Source: Lancet Glob Health. 2020 May 21;8(6):e829–39. doi: 10.1016/S2214-109X(20)30121-2 (PMC7248571; doi:10.1016/S2214-109X(20)30121-2)
Supplement: Supplementary appendix [file mmc1.pdf]

THE LANCET
Global Health

Supplementary appendix

This appendix formed part of the original submission and has been peer reviewed. We post it as supplied by the authors.

Supplement to: Watkins DA, Qi J, Kawakatsu Y, Pickersgill SJ, Horton SE, Jamison DT. Resource requirements for essential universal health coverage: a modelling study based on findings from *Disease Control Priorities*, 3rd edition. *Lancet Glob Health* 2020; **8:** e820–29.

Supplementary Appendix to "Costing a Priority-Based Package of Interventions for Universal Health Coverage in Low- and Lower-Middle-Income Countries: A Modelling Study"

Authors: David A. Watkins, Jinyuan Qi, Yoshito Kawakatsu, Sarah Pickersgill, Susan E. Horton, Dean T. Jamison

Table of contents

Overview of DCP3 essential packages and essential UHC 2
Table A1. DCP3's nine volumes 2
Figure A1. Development of the EUHC health benefits package 4
Costing approach 4
Data sources 6
Table A2. Summary of sources of cost data used in this model 6
Population in need 6
Baseline intervention coverage 7
Unit costs 7
Figure A2. Comparison of country-level health workforce salary estimates to GNI per capita estimates 9
Palliative care and pain control 9
Rehabilitation and disability 10
Pandemic preparedness 10
Essential pathology 10
Other unit cost components 10
Table A3. Calculation of above-facility cost markups using 2009 HLTF estimates 11
Sensitivity analysis 11
Table A4. Model parameters varied in sensitivity analysis 11
Figure A3. Results from one-way sensitivity analysis 12
Online visualisation tool 12
Additional results 13
Table A5. EUHC incremental costs and shares by intervention characteristic 13
Table A6. EUHC and HPP total costs and shares by health system objective 14
Table A7. Estimated current funding of EUHC as a percentage of estimated total costs 15
Table A8. Potential value for money from the HPP and EUHC 16
Comparison of DCP3 and other costing exercises 16
Table A9. Comparison of estimates from recent costing exercises 17
Table A10. Major differences between the DCP3 and WHO analyses of UHC costs 17
Table A11. Areas of discordance between the WHO and DCP3 intervention lists 18
Table A12. Countries included in the DCP3 and WHO analyses of UHC costs 19
References 21

Overview of DCP3 essential packages and essential UHC

Disease Control Priorities, Third Edition (DCP3) was a five-year collaborative effort among academics and practitioners working in global health to identify priorities for improving health in low-income countries (LICs) and middle-income countries (MICs). The DCP3 network comprised over 500 authors, 230 peer reviewers, and 33 editors who together produced 172 chapters across nine volumes (Table A1). These volumes were published over 2015-2018 by the World Bank, with each book volume and chapter available both in print and online (open access) at www.dcp-3.org. The key messages of DCP3 have been summarised in a series of *Lancet* reviews, including a capstone paper published in 2018.[1]

Table A1. DCP3's nine volumes

1. Essential Surgery – 2015
2. Reproductive, Maternal, Newborn and Child Health – 2016
3. Cancer – 2015
4. Mental, Neurological, and Substance Use Disorders – 2016
5. Cardiovascular, Respiratory, and Related Conditions – 2017
6. Major Infectious Diseases – 2017
7. Injury Prevention and Environmental Health – 2017
8. Child and Adolescent Health and Development – 2017
9. Disease Control Priorities: Improving Health & Reducing Poverty – 2018

Notes: Adapted from Jamison and colleagues[1]

Volume 9 summarised the recommendations of prior volumes in the areas of universal health coverage (UHC) and intersectoral policies for health, described the methodological advances in DCP3, and covered some cross-cutting themes, such as rehabilitation, pandemic preparedness, and palliative care. The costing exercise presented in this paper is built on a preliminary analysis of costs done for chapter 3 of volume 9, "Universal Health Coverage and Essential Packages of Care."[2]

The DCP3 project viewed the priority setting process through an economic lens. One of the primary outputs of DCP3 was a set of "essential packages" of health interventions. Each of the 21 essential intervention packages was oriented towards a particular professional community or set of related health topics. Intervention packages are lists of recommended health interventions (bundled by health topic) that seek to prevent, treat, cure, rehabilitate, or palliate one or more important causes of disease or injury in LICs and MICs.

Intervention packages were designed by the editors of each of DCP3's nine volumes in consultation with authors of specific chapters (i.e., topic experts). Three broad criteria were considered for including an intervention in an essential intervention package:

1. The intervention provides good value for money (usually in cost-effectiveness terms)
2. The intervention is feasible to implement in LICs and MICs
3. The intervention addresses a considerable disease burden in LICs and MICs

The sources of data reviewed in the development of the intervention packages included economic evaluations (often through original systematic reviews), clinical effectiveness studies, and epidemiological data (burden-of-disease estimates). Value for money was typically defined in terms of cost-effectiveness (cost per death or disability-/quality-adjusted life year) when these were relevant measures of value for money. Findings of DCP3's cost-effectiveness systematic reviews are summarised in chapter 7 of volume 9.[3] No specific cost-effectiveness "threshold" was provided to editors; however, as a general rule, only studies conducted in LIC/MIC settings were used to develop these packages. The feasibility criterion was implemented with reference to typical health system constraints and political economy considerations especially in LICs and lower-MICs.

To produce a unified and harmonised model health benefits package (HBP) for UHC systems, Watkins and colleagues extracted all of the **health sector** interventions contained in DCP3's 21 intervention packages. (Interventions that improve health but are implemented by other sectors, such as tobacco taxes [ministry of finance] and road safety measures [ministry of transportation] are dealt with in chapter 2 of DCP3 volume 9, "Intersectoral Policy Priorities for Health," and their costs are not included in this study.[4]) Watkins and colleagues then developed harmonised definitions of the health sector interventions (which included aggregating or disaggregating components in some cases), removed duplicates, and classified each intervention according to its delivery timing characteristic and the level of the health system (delivery "platform") at which it would typically be delivered. Chapter 3 of DCP3 volume 9 provides more details on this process.[2]

Regarding delivery timing characteristics, DCP3 conceived three levels of urgency of intervention delivery. **Urgent** interventions must be delivered quickly and close to where individuals live. Examples include basic district hospital surgical services (such as those required to manage injuries), management of acute coronary syndromes, and management of severe acute malnutrition. **Chronic** interventions do not have such an immediacy but require recurrent and frequent interactions between individuals with chronic illnesses and their healthcare providers – hence they must also be delivered close to where individuals live. Examples include antiretroviral therapy for HIV/AIDS and care for diabetes. **Time-bound (non-urgent)** interventions are generally "elective" (in clinical terms), allowing cases to be accumulated over space and time to generate efficiencies in delivery. Examples include immunisation campaigns and cataract repairs.

Regarding health system platforms, DCP3 developed a typology that included five distinct, logistically related delivery channels:

- **Population-based health interventions**: non-personal services typically organised by public health departments (such as media campaigns and vector control)
- **Community**: bringing health services close to where people live and work, mostly through sub-platforms like schools or community health workers
- **Health center**: clinics that deliver outpatient services (either comprehensive primary care centers or extension facilities like health posts)
- **First-level hospital**: a health facility that is able to provide inpatient care, including surgery; some specialty outpatient services (such as internal medicine and general surgery) are also included in this platform
- **Referral and specialised hospitals**: second- and third-level facilities that provide referral inpatient and highly specialised outpatient care (such as ophthalmology and urogynaecology services)

Health system arrangements vary greatly from country to country, so these platforms are inevitably highly stylised. Still, DCP3 argued that characterising interventions by these platforms would allow the user to conceptualise the integration of services and the costs required to deliver care at each level of the system.

The final, harmonised, deduplicated list contained 218 unique interventions, termed "essential UHC" (EUHC). The term EUHC was introduced as a distinction from the broader notion of UHC, which does not necessarily imply an explicit HBP. Yet the authors of DCP3 recognised that EUHC would constitute a demanding portfolio for many countries, especially LICs. To address these concerns, Watkins and colleagues developed a highest-priority package (HPP), defined as the subset of EUHC interventions that fulfilled the following criteria, balanced against each other:

1. **Very good value for money**. This was typically in cost-effectiveness terms as less than 0.5 times gross domestic product (GDP) per capita per DALY averted in a LIC, when DALYs were a relevant measure of health outcomes. (In cases like contraception, stillbirths, and palliative care, DALYs and QALYs are not relevant outcomes, so the more general assessment of "value for money" was used.)
2. **Priority to the worst off (in health terms)**. In a separate background paper for the forthcoming Lancet commission on reframing noncommunicable diseases and injuries for the poorest billion, Johannsen and colleagues describe the development of a metric that estimated lifetime health loss due to any particular cause.[5] For the purpose of this stylised package, the authors of DCP3 chose health conditions that reduce healthy life expectancy below 40 years as an indicator of "worst off" populations.
3. **Likely to offer substantial financial risk protection**. A composite indicator for financial risk protection was developed using unit cost and disease burden data. Interventions that, all else being equal, were very

costly (i.e., in the absence of public finance), addressed unexpected and acute illness, and addressed conditions that caused death or severe disability among wage-earning adults were deemed to be very likely to provide financial protection.

4. **Included in the "Grand Convergence" package.** This package was developed for the earlier *Lancet* Commission on Investing in Health.[6] These (predominantly reproductive, maternal, neonatal, and child health) interventions underwent careful scrutiny and economic analysis as a package and were generally included by default, though on the whole they fulfilled the criteria above as well.

Figure A1. Development of the EUHC health benefits package

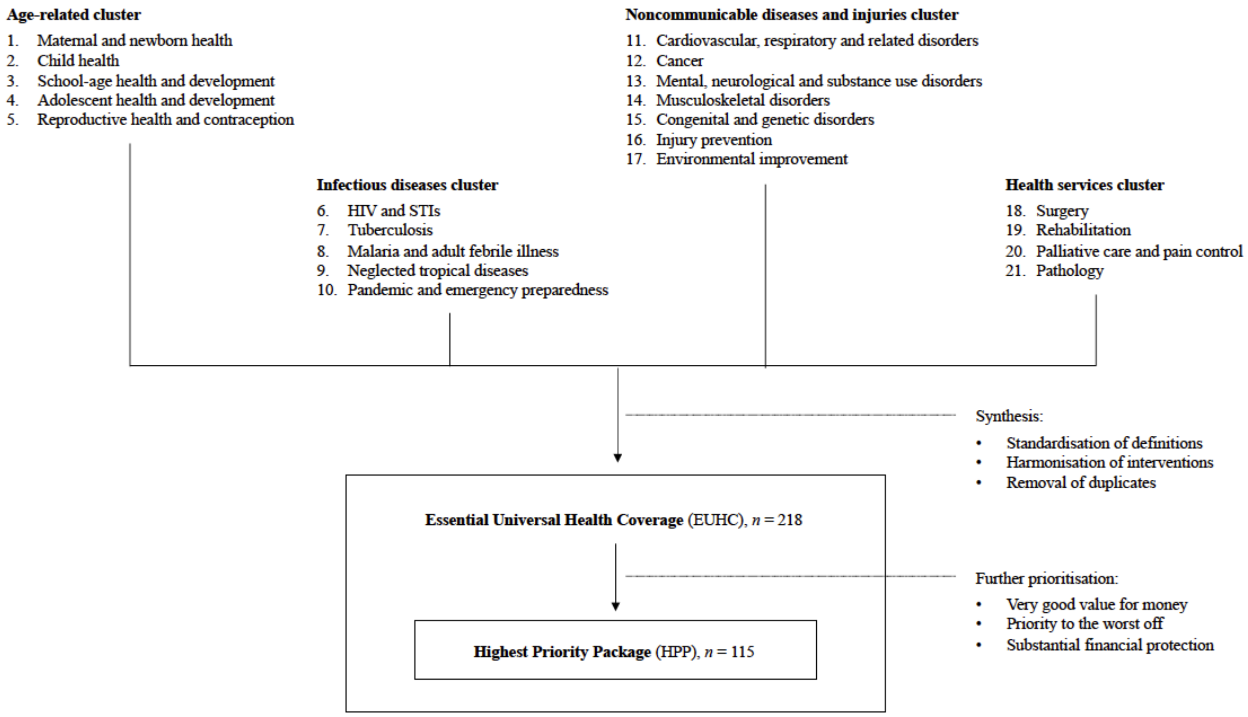

This figure shows how DCP3's 21 intervention packages were synthesised into the model health benefits packages, EUHC and HPP, that were costed in this study.

In the end, 115 of the 218 interventions fulfilled these criteria and were included in the model HPP. The costs of EUHC and the HPP subset are presented separately in this report.

Costing approach

As described in the main paper, the costing question we were trying to answer was, "how much (more) would typical LICs and lower-MICs be spending today if they had already fully implemented EUHC (or the HPP)?" Our costing approach was inspired by the comparative statics approach that is commonly used in economic analysis.[7] In our model, describe below, we treated population coverage of the interventions as an "exogenous" parameter and held constant all other parameters, such population size and structure and prices and quantities of goods and services. The resulting cost estimate would be interpreted as a counterfactual estimate of the difference in cost that would be expected from an instantaneous shift in coverage in the present day.

While this approach is a simplification of the potential costs in a given country, it was outside the remit of DCP3's analysis to systematically account for local contextual (health system) factors in a wide range of countries as well as develop sophisticated models that incorporate dynamic epidemiological and demographic effects of scaling up different interventions. Further, DCP3 was primarily concerned with the economic costs of interventions, usually as an input to economic evaluation and priority-setting. This perspective is fundamentally different from the approach

currently being used by WHO and the OneHealth Tool, which estimates incremental financial costs for other purposes such as budgeting and planning.[8]

Bearing these objectives in mind, we estimated counterfactual annual total and incremental costs without delving into variations in costs in specific countries or regions (which will inevitably deviate significantly from the stylised estimates we present).

The baseline cost (BC) of the EUHC package or HPP – i.e., the overall cost of all EUHC or HPP interventions as they are currently being implemented, can be represented simply as

$$\mathrm{BC} = \sum_{i=1}^{n} pop_i \times cov_{i,0} \times cost_i$$

where pop_i is a number of individuals in need of intervention i, $cov_{i,0}$ is the proportion of the population in need who are currently covered by intervention i (baseline coverage), and $cost_i$ is the unit cost of the intervention (incorporating both recurrent costs and annualised capital costs) per beneficiary (given by pop_i) served.

The total (counterfactual) present-day cost of EUHC or HPP at full implementation can be represented as

$$\mathrm{TC} = \sum_{i=1}^{n} pop_i \times cov_{i,1} \times cost_i$$

where $cov_{i,1}$ is the target proportion of the population in need who would be covered by intervention i (i.e., target coverage). For this analysis, we set $cov_{i,1}$ at 80% for all interventions, a target that is consistent with prior targets set by WHO for a variety of conditions and reflects a realistic target for most interventions during the SDG period.[9] Since each intervention is currently being delivered at a different coverage level, the coverage gap for each intervention will vary. Within this framework, the incremental (counterfactual) present-day cost of EUHC or HPP is

$$\mathrm{IC} = \mathrm{TC} - \mathrm{BC}$$

or alternatively,

$$\mathrm{IC} = \sum_{i=1}^{n} pop_i \times (cov_{i,1} - cov_{i,0}) \times cost_i$$

This approach requires unit cost estimates that reflect long-run average costs rather than marginal costs. An implication of using long-run average cost data is that cost structures will be more similar than different when extrapolated across regions (see below).

As described below, our unit cost data include both direct service delivery costs and health system costs. We used a series of markups to estimate health system costs for each intervention, and these costs were divided into facility-level "ancillary services" costs and above-facility costs:

$$cost_i = cost_{i,lit} + \alpha(cost_{i,lit}) + \beta\left[cost_{i,lit} + \alpha(cost_{i,lit})\right]$$

In this analysis, α and β are simply scalars derived from the literature; however, more complex functions (e.g., that vary by intervention scale, delivery platform, and/or programme area) could be specified.

In many countries, interventions that are offered through the public sector require significant cost-sharing or even cost-recovery through user fees. In these cases, our model would overestimate BC for an intervention at coverage level $cov_{i,0}$ from the health system perspective, since $cost_i$ would be partially borne by the user. Hence for these countries, within the context of costing achievement of UHC, the actual incremental cost IC' is likely to be higher than the estimated value IC, as IC' includes both the cost of going from $cov_{i,0}$ to $cov_{i,1}$ (described above) and the cost of reducing out-of-pocket payments for the proportion of the population currently covered at $cov_{i,0}$. It was

outside the scope of this costing exercise to generate precise, country-level estimates of CE and IC'; these would however be a necessary component of country-level priority setting and resource allocation exercise.

Data sources

The following sections detail data sources for costing most of the interventions in DCP3's 21 intervention packages. Methods and data sources for a few unique packages (rehabilitation, palliative care, and pandemics) are presented at the end of this section. Table A2 provides an overview of data sources for the three main sets of parameters in the cost model.

Table A2. Summary of sources of cost data used in this model

**Type of data**		**Main sources**
Population in need	Population estimates	UN Population Division estimates for 2015 (World Population Prospects 2017 update)[10]
	Population in need of reproductive health services	Scientific literature, including a 2011 Guttmacher Institute report[11]
	Incidence and prevalence of specific diseases addressed by EUHC interventions	Scientific literature, including the Global Burden of Disease 2016 Study and the WHO Global Health Observatory[12,13]
Baseline intervention coverage	Baseline coverage	WHO Global Health Observatory database of coverage indicators;[13] supplemented by scientific literature, expert opinion, or simple assumption in cases where published estimates or proxies were unavailable
Unit costs	Literature cost estimates	Unit costs taken from cost analyses in the scientific literature, giving weight to studies in low- and lower-middle-income countries; in a few cases we undertook our own cost analyses
	Nontraded proportion of unit costs	Generally assumed to be 70% based on review of a wide range of cost data; a few interventions that were predominately labor (e.g., rehabilitation services) or commodities (e.g., adding an antigen to EPI) had higher or lower assumed proportions
	Markups for ancillary services at the facility level	Indirect facility-level costs generally observed to be around 50% of service delivery costs[14]
	Markups for other health system costs above the facility level	Costs above the facility level reported to be about 17% of facility-level direct and indirect costs[15]

The online tool (see below) contains detailed data inputs and citations for each intervention. For all intervention-specific parameters citing "expert opinion," the experts consulted were selected from among the 78 co-authors of chapter 1 of volume 9 of DCP3, who served as technical experts for the formulation of DCP3's model HBPs (presented in this paper) and complementary list of intersectoral policies (described above and in chapter 2 of volume 9).[1]

Population in need

For most EUHC interventions, the "population in need" of the intervention was identified as the annual number of incident or prevalent cases of disease/injury for acute or ongoing (chronic) interventions, respectively. For some routine services, such as screening interventions, vaccinations, or family planning services, we used demographic estimates (e.g., 2015 birth cohort, women of reproductive age, adults 30-69 years, etc.). Often the epidemiological or demographic estimates were scaled down based on assumptions about the proportion of eligible individuals who would actually receive the service ("treated fraction"). For instance, screening for diabetes is recommended in adults over 40, but only every three years, so the number of individuals over 40 in the population would be divided by three to estimate the expected number of individuals in a given year who would receive screening.

Most epidemiological data were obtained as country-level "all ages" estimates of incidence or prevalence in the year 2015 from the Global Burden of Disease (GBD) 2016 study – unless similar data were available from the WHO – then aggregated into LIC and lower-MIC groups (see below).[12] For a variety of epidemiological estimates related to reproductive and maternal health, we used data from a report from the Guttmacher Institute on the cost of these services, which provided aggregate estimates by country income group.[11] Demographic estimates for the year 2015 were taken from the 2017 revision of the UN World Population Prospects.[10]

Our population estimates for LIC and lower-MIC countries (as groups) were based on our aggregation of country-level demographic estimates based on World Bank classification in 2014 (the list of countries by income group is provided in the section below that compares DCP3's cost estimates to those of WHO). The final estimates of aggregate population in 2015 were 0.90 billion for LICs and 2.7 billion for lower-MICs. We conducted a similar aggregation of gross national income (GNI) for countries for which GNI estimates were available (84% of LICs and 96% of lower-MICs, respectively). These values, when applied to the total population of those two income groups, were US$ 0.71 and US$ 5.9 trillion, respectively, or US$ 790 and US$ 2200 per capita respectively, in 2016 US dollars. Since GNI data were missing for some LICs, particularly countries that were fragile or in conflict (and likely poorer than average) our GNI figure for LICs could be an overestimate.

Baseline intervention coverage

Estimates of baseline coverage of specific interventions in LICs and lower-MICs are usually sparse. The WHO Global Health Observatory provides the most comprehensive list of coverage indicators, aggregated in many cases by country income group.[13] Where relevant and available, we used coverage indicators from WHO. For interventions that were closely related to a service for which we had coverage estimates, we assumed the available coverage estimate would be a reasonable proxy. (For instance, we have coverage rates for antiretroviral drug therapy but not for community-based HIV testing and counseling, so we assumed that coverage of testing and counseling would be similar to coverage of antiretrovirals.)

In a number of cases, particularly for some of the noncommunicable diseases (NCDs) and cross-cutting health systems interventions, we had no data on current coverage. Discussions with DCP3 authors and experts in LICs and lower-MICs supported the assumption that baseline coverage of these interventions would be very low. For interventions without good proxy indicators for coverage, we made the following assumptions: for Group I causes (communicable, maternal, perinatal, and nutritional disorders) we assumed baseline coverage of 40% and 50% in LICs and lower-MICs, respectively, which is similar to ANC4 (ie, the proportion of pregnant women attending four antenatal visits). For interventions addressing Group II and III causes (noncommunicable diseases and injuries) that did not have specific coverage indicators or reasonably proxies, we assumed baseline coverage of 5% and 8%, respectively, in line with the assumptions used in a CVD modeling study by WHO.[16] For cross-cutting packages (e.g., palliative care, rehabilitation), we assumed baseline coverage of 5% and 8% as well.

As mentioned previously, we chose 80% as the target coverage for all interventions. This suggests differential coverage gaps, ranging only a few percent for immunisations to nearly 75% for interventions for some neglected NCDs and cross-cutting health services. These gaps influence the estimates of incremental but not total costs. Implicit in this costing framework is that it is equally feasible by 2030 to address a 75% coverage gap for one intervention and a 5% coverage gap for another. In reality, there would be more momentum to close coverage gaps for major infectious diseases and maternal/child health interventions than for NCDs and injuries. DCP3 endorsed the progressive universalist approach to UHC as the most fair and efficient path, so we argue that if budget constraints are tight then a smaller set of interventions should be implemented at full coverage rather than a larger set at partial coverage.[6] As a result, the target coverage level of the HPP subset is the same (80%) as the target coverage level of EUHC.

Unit costs

We took as a starting point the unit cost estimates published in the cost and cost-effectiveness analyses included in systematic reviews undertaken for DCP3.[3,17-22] These reviews contain most of the highest-quality economic studies that have been conducted recently in LICs/MICs in their respective fields, and they include standard reporting measures for systematic reviews such as PRISMA flow diagrams and appraisals of study quality. Databases searched included Medline, Embase, NHS-EED, HEED (Cochrane), and Econlit, with terms for specific diseases,

terms for specific LIC and MIC (to capture studies done in any LIC/MIC), and terms related to economic analysis or evaluation. Brouwer and colleagues provide in their appendix an example of this search strategy for CVD costs.[23] We supplemented this database of economic evaluations with other studies cited in specific chapters of DCP3 or with our own literature search for intervention costs when we could not identify costs anywhere in DCP3. In a few cases where there were absolutely no previous published cost estimates, we undertook our own "bottom-up" costing using assumptions about personnel, equipment, and drugs and consumables (all detailed in the online tool described below).

For a number of areas (such as HIV, TB, and child health interventions), multiple unit cost data points were available and were included in the DCP3 reviews. To guide the selection of one unit cost data point for each intervention, we employed the following criteria (balanced against each other):

1. Degree of similarity (e.g., specific inputs and activities) between the intervention being costed in the study and the intervention recommended by DCP3
2. Study quality -- DCP3's systematic reviews used a quality scoring rubric adapted from the Drummond checklist.[24] We made use of this information when available; when not available, our judgment of quality was based on the degree of detail included in the methods and results sections of the paper (with reference to the specific unit cost we were seeking)
3. All else equal, more recent studies, and studies originating from low- or lower-middle-income countries, were given preference.

We gave preference to studies from LICs and lower-MICs, but we used upper-MIC country data when necessary. It could be argued that cost structures for interventions across these country settings would vary substantially. We sought to identify estimates of long-run average costs, which would theoretically mitigate this concern partially – in the case of long-run average costs, all cost components would be variable costs. Two significant sources of variation in cost structure across country settings would remain: regional practice patterns that result in different quantities of resources consumed, and systematic differences in price structures across regions due to tariffs or other policies; accounting for both of these sources of variation was outside the scope of this costing exercise.

In all cases, we represent costs in 2016 US dollars. This required us to convert and inflate nearly all unit cost estimates. To accomplish this, we used the procedure recommended by the Global Health Cost Consortium: (i) convert published estimate to local currency units (in the year study data were collected), (ii) split the cost into traded and non-traded components (see below), (iii.a) inflate the traded component of the cost to 2016 using the global inflation rate and (iii.b) inflate the nontraded component of the cost to 2016 using local consumer price index data then (iv) convert from local currency units to US dollars using mid-2016 exchange rates. Exchange rate, consumer price index, PPP, and regional inflation data were taken from the 2018 World Development Indicators and World Economic Outlook database (April 2019 revision).

For some interventions, the only or best unit cost data were reported as averages by World Bank income group. There is lack of clarity in the literature on the best approach to inflating costs in groups of countries. Three general approaches have been proposed: using the inflation rate for the median country in the group, conducting a principal component analysis of country panel data on inflation and using the first principal component as a measure of group inflation, and using the average country inflation rate, weighted by GDP.[25] We chose the third approach (average country inflation rate, weighted by GDP), since this approach would most easily replicable with updated GDP estimates in subsequent years, and it would be straightforward to implement.

We modified our unit cost data to account for price differences between country income groups. To accomplish this, we employed the assumption that prices of non-traded goods and services would be roughly proportional to GNI per capita when compared across countries and regions. The estimate of the non-traded component of $cost_{i,y}$ (the unit cost of the ith intervention in country income group Y) would be expressed as

$$cost_{i,Y,nontraded} = \left[\delta \times \left(cost_{i,x}\right) \times \frac{\bar{s}_Y}{\bar{s}_x}\right] + \left[(1-\delta) \times \left(cost_{i,x}\right)\right]$$

where δ is the proportion of healthcare costs that are non-traded, $cost_{i,x}$ is the "raw" unit cost estimate for intervention i in country x, $\bar{s}_Y$ is the population-weighted average GNI per capita in country income group Y, and $\bar{s}_x$

is the GNI per capita in country x. For most interventions, we assigned δ a value of 0.70, which is an average value based on analyses of WHO System of Health Accounts data. For interventions that had no traded component, such as physical therapy services, we assigned δ a value of 1.0, and for interventions that were only commodities (e.g., some of the vaccine antigens, incremental to EPI), we assigned δ a value of 1.0. As described above, traded and non-traded components were inflated using global vs. country/regional inflation, respectively.

The assumption that non-traded healthcare costs vary proportionally to GNI per capita was tested by comparing health worker salaries to GNI per capita (Figure A2).

Figure A2. Comparison of country-level health workforce salary estimates to GNI per capita estimates

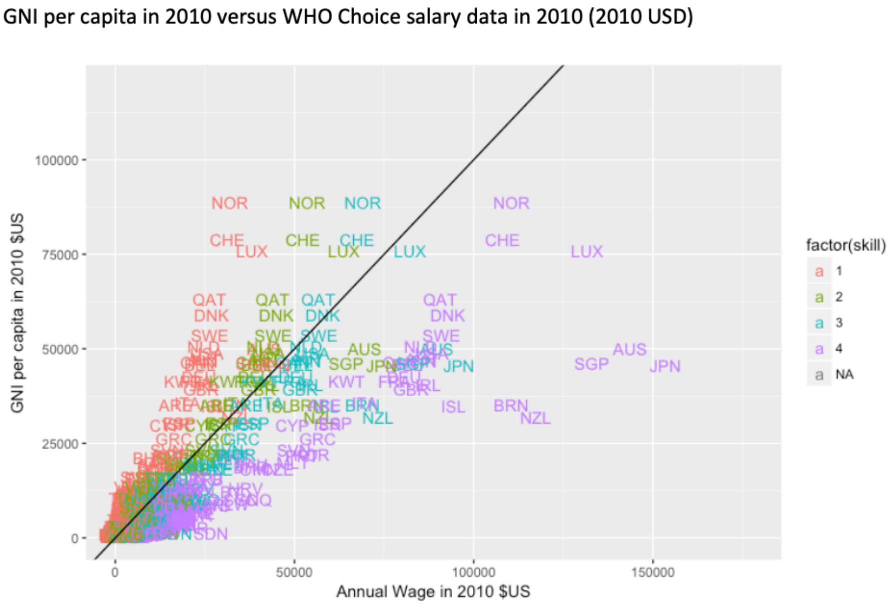

Note: colours indicate different skill levels (red = least skilled; purple = most skilled). Data taken from WHO-CHOICE (with appreciation to J. Serje, 2015).

As can be seen above, health worker salaries are correlated with GNI per capita across countries, though the strength of the relationship and its deviation from a 1:1 relationship vary by skill level. Since most health workers in LIC and MIC settings are lower-skilled (level 1-3 in the WHO-CHOICE classification system), we think it is unlikely that our extrapolation approach (shown by the equation above and black line in Figure A1) significantly underestimates labour costs.

A few intervention packages from volume 9 of DCP3 deviated from our general costing approach by virtue of their delivery properties:

Palliative care and pain control

Costs for the palliative care essential package were estimated in chapter 12 of volume 9 of DCP3.[26] The authors of that chapter, representing the Global Alliance for Palliative Care and Pain Control, conducted a bottom-up costing of palliative care services using detailed data from three countries. They concluded that the package would cost about US$ 2.6 and US$ 0.82 per capita annually in typical LICs and lower-MICs, respectively. The basic components of their package were very similar to the interventions recommended in DCP3, but they did not report unit costs for each "intervention." Hence for this study we took the package costs as a whole. Based on expert opinion, we assigned the HPP palliative care intervention ("essential palliative care and pain control measures, including oral immediate release morphine and medicines for associated symptoms") 25% of the total cost of the package.

Rehabilitation and disability

The package of rehabilitation "interventions" presented in DCP3 is oriented around meeting target levels of human resources rather than scaling up particular equipment, drugs, and consumables. Further, other costs beside human resources are likely to vary widely according to epidemiological context. For instance, older populations suffering from causes like stroke or visual/hearing impairment will require more assistive devices, whereas younger populations suffering from causes like injury will require more rehabilitative exercise equipment. WHO has recommended a target of 750 rehabilitation specialists per million population, so we estimated the cost of the rehabilitation package as the cost of these human resources. We used WHO-CHOICE data on salary levels for skilled healthcare workers to generate a proxy unit cost of the package. We assumed that most rehabilitation specialists would be classified as skill level 3 in the WHO-CHOICE classification system. No components of this package were included in the HPP.

Pandemic preparedness

The pandemic preparedness package largely followed the recommendations of the Commission on a Global Health Risk Framework for the Future, which drew on costs estimated by a 2012 World Bank report for scaling up preparedness in a large number of low- and middle-income countries.[27] This report estimated that the annual incremental cost of bringing all LICs and MICs up to international preparedness standards would be between US$ 1.9 billion and US$ 3.4 billion (in 2012 US dollars) depending on epidemiological assumptions. We selected the higher cost and applied it on a per-capita basis in our two country income groups. No components of this package were included in the HPP.

Essential pathology

The human and material resources outlined in this package largely followed on from the interventions DCP3 defined as "essential." For instance, the package included point-of-care diagnostic tests for major infectious diseases and common NCDs at lower-level facilities, and these diagnostics generally aligned with the interventions for major infectious disease and NCDs outlined in the other essential packages. The typical cost of such a package of pathology services was estimated to be 6% of the total service delivery costs (i.e., of essential clinical interventions).[28] We included this markup in our estimates of facility-level "ancillary services" (described below) but reported this component of indirect cost as a unique package (i.e., separate from the "total health system costs" in the main manuscript's table of costs by package). The same markup of 6% was applied both to the EUHC and to the HPP sets of interventions.

Other unit cost components

In addition to the costs of service delivery, derived largely from the literature, we incorporated other health system costs that would be required to scale up and support essential interventions. We broke down the non-service delivery costs into two components: facility-level costs due to "ancillary" services and "above-facility" health system costs. Facility-level costs include all health facility costs that are not directly allocable to specific clinical services but are required in order for those services to be delivered. Examples include pathology, radiology, administration, maintenance, and so on. The costs of these ancillary services are not usually included in microcosting studies such as the ones we used to derive intervention unit costs. We applied a standard markup of 50% to our estimated service delivery costs.[14] This figure is in line with analyses of WHO System of Health Accounts data that one of our co-authors (S. Horton) has done on the overall magnitude of healthcare costs that are not allocable to specific health services. As noted above, pathology services are defined as part of facility-level costs, so we broke down this markup into 6% for the pathology component (i.e., the essential pathology package) and 44% for all other components.

Above-facility costs are for those aspects of health systems that support disease-specific programs but (i) cannot be allocated to specific interventions (similar to ancillary services) and (ii) occur outside of healthcare delivery facilities. The 2009 WHO High Level Taskforce (HLTF) on innovative international financing for health systems explored the relative magnitude of these costs for Millennium Development Goals interventions.[15] That report identified four unique system cost categories that were not accounted for service delivery costs or facility-level costs

discussed above: supply chain/logistics, health information systems, governance, and health financing. We averaged out the ranges of shares of incremental costs across four scenarios ("WHO normative" and three variants based on the Marginal Budgeting for Bottlenecks approach), then converted these shares to markups (Table A3). For example, if health system costs were 14% of incremental costs, then they would be estimated as 0.17 times the value of the non-health-system costs.

Table A3. Calculation of above-facility cost markups using 2009 HLTF estimates

Category	WHO normative	MBB minimum	MBB medium	MBB maximum	Average	Minimum	Maximum
Supply chain/logistics	5%	5%	8%	9%	6.8%	5%	9%
Health info. systems	2%	2%	1%	1%	1.5%	1%	2%
Governance/admin	2%	6%	6%	3%	4.3%	2%	6%
Health financing	4%	0%	2%	1%	1.8%	0%	4%
				Total	14%	8%	21%
				Markup factor	0.17	0.09	0.27

Sensitivity analysis

We conducted both one-way and probabilistic sensitivity analyses as described in the main manuscript. Model parameters varied in the sensitivity analyses are shown in Table A4 below. Due to the large number of interventions, it was computationally burdensome to generate random values for each parameter for each intervention, so we did 10,000 Monte Carlo simulations drawing one value for each parameter and applying it to all interventions. Because uncertainty was calculated using multipliers (scalars) of base case values rather than by constructing "natural" distributions for each intervention-parameter set (e.g., gamma distribution for unit cost), we assumed a triangular distribution for all parameters. We estimated the uncertainty of the summary indicators (incremental and total cost) by extracting the values of quantiles 0.025 and 0.975 from their final distributions.

Table A4. Model parameters varied in sensitivity analysis

Parameter	Base value	Low value	High value	Rationale for plausible range
Unit cost multiplier	1.00	0.70	1.30	Triangulation of empirical evidence and standard practice in economic evaluations (see footnote)
Population in need multiplier	1.00	0.90	1.10	Average 95% uncertainty interval for global prevalence estimates for common causes (e.g., HIV, IHD) in GBD 2017
Baseline coverage adjustment	0.00	-0.15	0.15	Variation in WHO's "service coverage index" across LICs and MICs with "low" or "medium" data availability
Markup for facility-level (ancillary services) costs	0.50	0.30	0.80	Range of variation in expenditures that cannot be allocated to disease-specific activities (SHA 2011 analysis by S. Horton)
Markup for above-facility costs	0.17	0.09	0.27	Range of incremental health system costs across 4 scenarios in 2009 HLTF (see Table A3)
Total fertility rate (LICs) multiplier	1.00	0.87	1.10	Ratio of projected TFR in 2025-2035 (low or high vs. medium variant, respectively) by income group, as per WPP 2017
Total fertility rate (lower-MICs) multiplier	1.00	0.80	1.20	Ratio of projected TFR in 2025-2035 (low or high vs. medium variant, respectively) by income group, as per WPP 2017

Note: each of the model inputs was multiplied by the respective parameter in the table above, except in the case of the coverage gap, which was added. For example, in the base case, the unit costs were multiplied by 1.0, and values between 0.50 and 1.50 were used in the sensitivity analyses, and so on for the other parameters; for baseline coverage, 0% was added in the base case and values between -10% and +10% were subtracted or added, respectively, in the sensitivity analysis. The choice of plausible range for unit costs was informed by a study of ART costs in

Nigeria.[29] In their sample of 80 health facilities, the ratio q75/q25 of unit cost of ART was a factor of 1.84, which corresponds roughly to +/- 30% of the mean value. (We chose not to use their maximum/minimum ratio, a factor of 22. Part of their study's objective was to demonstrate the wide range of empirical unit costs rather than quantify normative variation in unit costs in an ideal implementation scenario, the latter of which is closer to what we are analysing in this paper.) We also note that cost-effectiveness analysis studies often assume unit costs vary by +/- 33% in the absence of empirical evidence of statistical uncertainty.

Figure A3 provides results from the one-way sensitivity analysis. The largest *quantitative* drivers of uncertainty in our model are unit cost estimates. This finding underscores the need for high-quality empirical data on costs in general—particularly when moving from a global exercise like this one to a country-specific exercise that involves spending of real money.

The uncertainty in baseline coverage, while significant, does not reflect the reality that, in many cases, coverage levels were assigned based on expert opinion or assumption. It is possible that +/- 15% uncertainty in baseline coverage is simply too conservative, though we have no empirical basis or standard practice upon which to choose an alternative range of values. Apart from unit cost data collection, routine and standardised approaches to estimating coverage of a wide range of health interventions (including NCDs and injuries) is needed both to gauge progress on UHC and improve the accuracy and precision of incremental costs estimates in this study and others.

Figure A3. Results from one-way sensitivity analysis

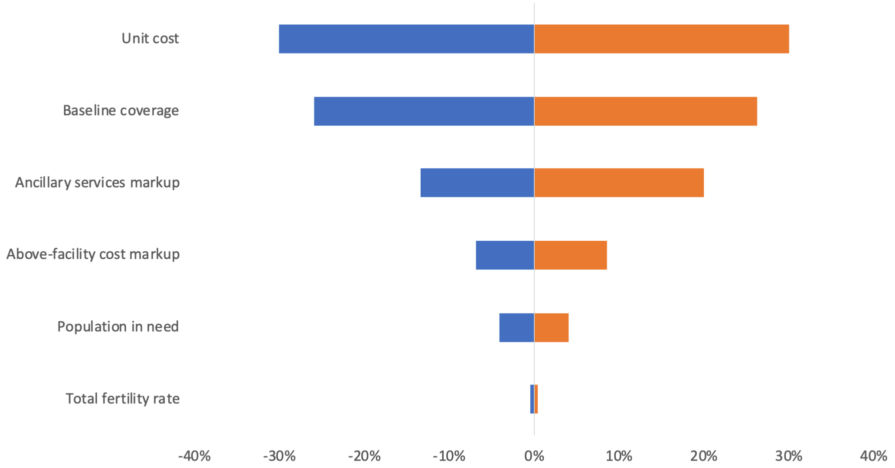

The tornado plot shows the variation in incremental annual cost (for low- and lower-middle-income countries combined) resulting from changing model parameters to their upper (orange) or lower (blue) plausible ranges, one at a time (ranges are listed on p. X of the appendix). "Variation in fertility rate" is the additional uncertainty related to UN Population Division short-term projections of total fertility rate – above and beyond the uncertainty in the "population in need" for reproductive health interventions. This parameter only affects the populations in need of reproductive, maternal, and perinatal health interventions in 2015 as we do not incorporate future demographic changes into our model.

Online visualisation tool

As described in the text, we developed a web-based version of the model (https://dcp-uw.shinyapps.io/dcp-cm/) to allow readers to inspect the data sources and assumptions that go into each intervention. The tool also has an editable table function that allows the reader to change any data input value for any intervention (or combination of interventions); edited model inputs are the used to re-calculate health benefit package costs. This online visualisation tool was developing using RShiny. Source code for the RShiny app and data input files (e.g., used for currency conversion) are available on Github (link provided in the Shiny app).

The online tool is not designed to generate precise estimates of the cost of interventions to do budgeting and planning at a country level. Rather, it is meant to give the user a sense of the probable magnitude of the cost of various combinations of interventions (or EUHC as a whole) in two country economic contexts (LIC and lower-MIC) that in reality are very heterogeneous mixes of countries and health system arrangements. The tool should be understood as primarily an academic effort to produce generic estimates for broad audiences and stimulate dialogue on UHC financing and priority setting.

In principle, a user who is working in a particular LIC or lower-MIC could modify the selection of interventions and the model parameters in this tool to generate crude local cost estimates – perhaps as a starting point for identifying potentially high- or low-cost interventions or high-priority areas to do primary data collection. In other words, the tool might in some instances be useful early in a country's policy cycle as an input to agenda-setting and policy formulation, but not as a guide to decision-making or planning.

Additional results

Table A5 provides a breakdown of incremental costs by (i) intervention temporal characteristics and (ii) delivery platform, including 95% credible intervals.

Table A5. EUHC incremental costs and shares by intervention characteristic

			**Population-based**	**Community**	**Health center**	**First-level hospital**	**Referral and specialty hospitals**
**Urgent**	*LICs*	cost (US$, billions)	$0.0<br>(0.0 – 0.0)	$1.5<br>(0.96 – 2.3)	$2.9<br>(2.0 – 4.2)	$13<br>(9.6 – 19)	$0.49<br>(0.36 – 0.69)
		share of overall cost	0.0%<br>(0.0 – 0.0)	2.7%<br>(2.2 – 3.0)	5.1%<br>(4.8 – 5.3)	23%<br>(23 – 24)	0.86%<br>(0.82 – 0.90)
	lower-MICs	cost (US$, billions)	$0.0<br>(0.0 – 0.0)	$3.1<br>(1.2 – 5.8)	$8.0<br>(5.1 – 12)	$56<br>(39 – 79)	$1.7<br>(1.2 – 2.4)
		share of overall cost	0.0%<br>(0.0 – 0.0)	1.2%<br>(0.62 – 1.8)	3.2%<br>(2.8 – 3.5)	23%<br>(22 – 23)	0.68%<br>(0.64 – 0.73)
**Chronic**	*LICs*	cost (US$, billions)	$0.0<br>(0.0 – 0.0)	$1.4<br>(1.0 – 1.9)	$20<br>(14 – 28)	$1.6<br>(1.2 – 2.3)	$0.50<br>(0.36 – 0.69)
		share of overall cost	0.0%<br>(0.0 – 0.0)	2.4%<br>(2.3 – 2.5)	35%<br>(34 – 36)	2.8%<br>(2.7 – 2.9)	0.87%<br>(0.85 – 0.90)
	lower-MICs	cost (US$, billions)	$0.0<br>(0.0 – 0.0)	$8.6<br>(6.2 – 12)	$95<br>(67 – 130)	$8.8<br>(6.3 – 12)	$2.5<br>(1.8 – 3.5)
		share of overall cost	0.0%<br>(0.0 – 0.0)	3.5%<br>(3.3 – 3.7)	39%<br>(38 – 39)	3.6%<br>(3.4 – 3.8)	1.0%<br>(0.97 – 1.1)
**Time-bound (non-urgent)**	*LICs*	cost (US$, billions)	$1.2<br>(0.81 – 1.7)	$4.4<br>(3.0 – 6.5)	$5.6<br>(4.0 – 7.9)	$2.7<br>(1.9 – 3.7)	$1.6<br>(1.1 – 2.2)
		share of overall cost	2.0%<br>(1.9 – 2.1)	7.8%<br>(7.2 – 8.3)	9.8%<br>(9.6 – 9.9)	4.7%<br>(4.6 – 4.9)	2.7%<br>(2.6 – 2.9)
	lower-MICs	cost (US$, billions)	$3.6<br>(2.4 – 5.2)	$17<br>(11 – 25)	$20<br>(14 – 28)	$12<br>(8.8 – 17)	$11<br>(7.7 – 15)
		share of overall cost	1.5%<br>(1.4 – 1.5)	6.9%<br>(6.5 – 7.2)	8.1%<br>(7.9 – 8.3)	4.9%<br>(4.7 – 5.3)	4.3%<br>(4.1 – 4.7)

Table A6 provides a breakdown of total costs by health system objective, including 95% credible intervals (not provided in manuscript Table 3 due to space constraints).

Table A6. EUHC and HPP total costs and shares by health system objective

	Total	Mortality reduction; under-5	Mortality reduction: age 5+, group I causes	Mortality reduction: age 5+; group II-III causes	Reduction in disability	Non-health outcomes
**HPP**						
Number of interventions	115	38	18	27	15	12
LICs - total cost (% GNI)	5.1% (3.8 – 6.8)	1.4% (1.1 – 1.9)	0.8% (0.6 – 1.0)	1.3% (1.0 – 1.7)	1.0% (0.7 – 1.3)	0.60% (0.5 – 0.8)
LICs - total cost (US$ [bn] / year)	36.1 (27.2 – 48.1)	10.1 (7.6 – 13.5)	5.6 (4.2 – 7.4)	9.0 (6.8 – 12.0)	7.0 (5.3 – 9.2)	4.5 (3.4 – 6.0)
LICS - share of overall costs	100%	28.0% (27.5 – 28.6)	15.4% (15.2 – 15.6)	24.9% (24.5 – 25.3)	19.3% (19.0 – 19.6)	12.4% (12.3 – 12.5)
Lower-MICs - total cost (% of GNI)	3.1% (2.3 – 4.1)	0.7% (0.6 – 1.0)	0.4% (0.3 – 0.5)	1.0% (0.8 – 1.4)	0.7% (0.6 – 1.0)	0.2% (0.2 – 0.3)
Lower-MICs - total cost (US$[bn] / year)	183.3 (138.3 – 244.6)	43.2 (32.6 – 57.7)	20.6 (15.4 – 27.5)	61.6 (46.4 – 82.4)	43.6 (32.9 – 57.9)	14.4 (10.8 – 19.1)
Lower-MICs-share of overall costs	100%	23.6% (23.2 – 24.1)	11.2% (10.9 – 11.5)	33.6% (33.3 – 33.9)	23.8% (23.4 – 24.1)	7.8% (7.8 – 7.9)
**EUHC**						
Number of interventions	218	52	44	45	58	19
LICs - total cost (% GNI)	10.0% (7.5 – 13.0)	1.6% (1.2 – 2.2)	1.6% (1.2 – 2.1)	3.7% (2.8 – 5.0)	2.2% (1.7 – 3.0)	0.8% (0.6 – 1.0)
LICs - total cost (US$ [bn] / year)	71.0 (53.5 – 94.6)	11.5 (8.7 – 15.4)	11.4 (8.6 – 15.0)	26.7 (20.1 – 35.4)	15.8 (11.9 – 21.1)	5.6 (4.2 – 7.4)
LICS - share of overall costs	100%	16.2% (16.0 – 16.5)	16.1% (15.7 – 16.4)	37.6% (37.2 – 37.9)	22.3% (22.3 – 22.3)	7.8% (7.8 – 7.9)
Lower-MICs - total cost (% of GNI)	6.0% (4.5 – 8.0)	0.9% (0.7 – 1.2)	0.8% (0.6 – 1.0)	2.6% (1.9 – 3.4)	1.5% (1.1 – 2.0)	0.3% (0.2 – 0.4)
Lower-MICs - total cost (US$[bn] / year)	353.9 (267.1 – 471.7)	51.0 (38.4 – 68.0)	44.4 (33.3 – 59.4)	152.3 (115.0 – 202.6)	88.9 (67.0 – 118.3)	17.4 (13.1 – 23.2)
Lower-MICs-share of overall costs	100%	14.2% (14.2 – 14.7)	12.5% (12.2 – 12.9)	43.0% (42.6 – 43.4)	25.1% (25.0 – 25.2)	4.9% (4.9 – 4.9)

We also looked at the relative "underfunding" of different components of the UHC packages and of the packages on the whole. Underfunding reflects the ratio of current spending to total cost at full (80%) coverage, ie:

$$\%\ \text{underfunded}_i = \frac{(\text{TC}_i - \text{IC}_i)}{\text{TC}_i}$$

Values closer to zero indicate relative under-prioritisation, whereas values closer to one indicate that levels of investment are approaching levels needed to deliver EUHC. Table A7 presents these quantities by package, intervention characteristic, and income group.

Table A7. Estimated current funding of EUHC as a percentage of estimated total costs

	Low-income countries	Lower-middle-income countries
**By benefit package**		
EUHC	19.4%	30.3%
HPP	26.2%	36.7%
**By platform**		
Population-based	33.6%	41.5%
Community	42.0%	48.4%
Health center	17.1%	28.9%
First-level hospital	9.6%	25.7%
Referral and specialty hospitals	3.5%	8.6%
**By temporal characteristics of intervention**		
Urgent	20.9%	39.3%
Chronic	13.1%	24.4%
Time-bound (non-urgent)	26.0%	29.0%
**By essential intervention package**		
Age-related		
1. Maternal and newborn health	40.9%	43.3%
2. Child health	67.0%	74.6%
3. School-age health and development	33.3%	30.5%
4. Adolescent health and development	41.3%	41.3%
5. Reproductive health and contraception	65.7%	78.8%
Infectious diseases		
6. HIV and STIs	36.0%	21.6%
7. Tuberculosis	70.1%	63.7%
8. Malaria and adult febrile illness	38.2%	64.5%
9. Neglected tropical diseases	26.1%	32.6%
10. Pandemic and emergency preparedness	2.1%	10.0%
Noncommunicable disease and injury		
11. Cardiovascular, respiratory and related disorders	5.5%	31.7%
12. Cancer	6.9%	13.9%
13. Mental, neurological, and substance use disorders	8.5%	8.6%
14. Musculoskeletal disorders	3.6%	7.7%
15. Congenital and genetic disorders	30.0%	23.7%
16. Injury prevention	4.2%	3.2%
17. Environmental improvement	47.1%	66.6%
Health services		
18. Surgery	5.3%	11.8%
19. Rehabilitation	6.3%	10.0%
20. Palliative care and pain control	6.3%	10.0%
21. Pathology	17.1%	17.8%

In Table A8, we look at potential value for money that could be realised by implementing the two packages at 80% coverage by 2030. The Table was constructed using two different data sources: incremental costs (from this study) and estimated incremental deaths averted for the year 2030 (taken from Table 1 of Jamison and colleagues).[1]

Table A8. Potential value for money from the HPP and EUHC

	Low-income countries		Lower-middle-income countries	
	HPP	EUHC	HPP	EUHC
**Under-5 deaths**				
Incremental cost	$5.0	$5.8	$15	$18
Deaths averted	0.62	0.77	1.1	1.3
Cost per death averted	$8100	$7500	$14 000	$14 000
**Deaths age 5-69, group I causes**				
Incremental cost	$3.4	$7.7	$10	$29
Deaths averted	0.59	0.65	0.85	0.94
Cost per death averted	$5800	$12 000	$12 000	$31 000
**Deaths age 5-69, group II-III causes**				
Incremental cost	$8.6	$25	$40	$110
Deaths averted	0.40	0.59	1.4	2.0
Cost per death averted	$22 000	$42 000	$29 000	$55 000

Notes: Incremental costs are in billions of 2016 US dollars. Estimates of deaths averted are in millions of deaths. Group I causes are communicable, maternal, perinatal and nutritional conditions; group II causes are noncommunicable diseases; group III causes are injuries. HPP = highest priority package. EUHC = essential universal health coverage.

Both analyses used the same combinations of interventions and the same assumptions about baseline and target intervention coverage, so incremental annual costs in this study can reasonably be compared to incremental annual mortality benefits to get a sense for value for money of the two UHC packages. *This Table does not, however, constitute a formal cost-effectiveness analysis.*

Comparison of DCP3 and other costing exercises

We compared our estimates to several published "price tag" analyses that had some degree of comparability in terms of interventions and/or countries included. Table A9 provides a summary of these findings. Overall, our estimated incremental cost of EUHC of US$ 310 billion annually across all LIC and lower-MIC countries is most similar to what was estimated by Stenberg and colleagues in 2017 for their UHC price tag study (US$ 370 billion annually). Since their study included upper-MICs and ours did not, it is not surprising that their costs are higher; however, because they costed fewer interventions, particularly for NCDs and injuries (which are often more expensive), it is also not surprising that their estimates are not higher by a large factor.

Roughly speaking, if we assume demography and epidemiology are similar between lower-MICs and upper-MICs, most of EUHC costs are proportional to country income, and the coverage gap in upper-MICs is about half of that of lower-MICs, we might get an incremental cost on the order of US$ 300-400 billion for upper-MICs, which when added to our estimate of US$ 310 billion for LICs and lower-MICs, would translate into US$ 600-700 billion for all LICs and MICs combined. A future version of our model will include inputs for upper-MICs.

Table A9. Comparison of estimates from recent costing exercises

Author/source	Interventions/targets	Country groups	Annual incremental cost	Currency -year
**Stenberg[8]**	all conditions, all health system levels	67 LIC and MIC	$371 billion annually by 2030	2014 USD
**Stenberg[30]**	all conditions, PHC only	67 LIC and MIC	$200 billion annually over 2020-2030	2014 USD
**Jamison[6]**	RMNCH, HIV, TB, malaria, NTDs	34 LIC and 3 large LMIC extrapolated to 82 LIC + lower-MIC	$27 billion annually at higher scale (2026-2035)	2011 USD
**Stenberg[31]**	RMNCH	74 high-burden countries (LIC and MIC, including India and China)	$30 billion annually by 2035	2011 USD
**Schwartlander[32]**	HIV/AIDS control	139 LICs and MICs	$22 billion in 2015, assuming universal coverage achieved	not specified
**Reid[33]**	TB elimination	all countries	$5 billion annually (medium term)	not specified
**Feachem[34]**	Malaria control and elimination	all countries	$8.7 billion annually by 2030	2014 USD
**Bertram[16]**	CVD	20 high-burden countries	$8 billion annually during SDG period	2015 USD

We also validated our estimates of baseline costs using estimates of current spending in LICs and lower-MICs. For all the countries included in our study, we extracted data from the WHO's Global Health Expenditure Database (accessed 5 Mar 2020) regarding current population, current health expenditure, and the share of current health expenditure due to external sources and general government health expenditure from domestic sources. Public spending on health – defined as government health expenditure from both domestic and external sources combined – was US$ 18 per capita in LICs and US$ 39 per capita in lower-MICs (2017 US$). By comparison, we estimated the baseline cost of EUHC to be US$ 14 and US$ 23, respectively, a reasonable fraction of overall public spending. (It is not surprising that baseline EUHC costs are a greater share of total public spending in LICs. We would expect health sector investments in LICs to more closely mirror the sorts of interventions in EUHC and include fewer interventions outside EUHC; whereas in lower-MICs, public spending is probably being devoted to a wider range of non-EUHC investments, such as NCD interventions.)

Finally, we did a more in-depth comparison of DCP3's estimates to those of Stenberg and colleagues (2017 global price tag paper for achieving the health-related SDGs).[8] Their analysis used the OneHealth Tool to calculate the incremental financial costs of scaling up health system building blocks (e.g., health facility density, health workforce density) to target levels in 67 representative LICs and MICs (including some upper MICs). Their estimates also included the incremental costs of 187 health interventions that would be supported by these enhanced health system resources. Table A10 outlines the major differences between the two sets of analyses, and Table A11 outlines the differences between the two lists of interventions analysed.

Table A10. Major differences between the DCP3 and WHO analyses of UHC costs

Study feature	DCP3	WHO
**Types of costs presented**	Total and incremental (counterfactual) costs in 2015 assuming instantaneous shift in coverage to 80%	Incremental yearly costs through 2030; total cost in 2030 extrapolated based on current expenditure
**Selection of interventions**	218 interventions recommended by technical experts (DCP3 authors and editors)	187 interventions recommended by WHO disease-specific clusters

**Scenarios assessed**	**1. Essential UHC (EUHC)** = sum of all recommended health sector interventions in DCP3<br>**2. Highest-priority package (HPP)** = narrower scope (108 interventions) compared to EUHC (prioritised on the basis of explicit criteria); same target coverage level (80%)	**1. Progress** = target coverage limited by absorptive capacity of system (target coverage levels vary by country and intervention type)<br>**2. Ambitious** = most countries achieve high levels of target coverage (and hence SDG3 coverage and mortality targets)
**Inclusion of costs of non-health sector interventions**	Not included	Included, but only with health sector component of costs ("above the line")
**Analytic tool(s) and cost data**	R-based (including RShiny-based tool for users). Unit costs using a bottom-up approach were taken from the literature and adjusted to "average" LIC/lower-MIC country prices.	OneHealth Tool, with some modeling in Excel. All unit costs were calculated using a bottom-up approach based on OneHealth Tool assumptions and WHO-CHOICE price databases

Table A11. Areas of discordance between the WHO and DCP3 intervention lists

**Included by DCP3 but not by WHO**	**Included by WHO but not by DCP3**
More detailed reproductive health interventions (unable to compare directly with "family planning" intervention per WHO)	Iodine supplementation for pregnant women and children
More detailed surgical interventions (unable to compare directly with "surgical and trauma care" intervention per WHO)	Basic antenatal care (4 visits) (captured in DCP3's detailed costing of antenatal interventions, but not an explicit intervention itself)
Treatment of childhood cancers	Cash transfers to poor women to deliver in facilities (regarded as an access intervention in DCP3, not a specific health service)
Adolescent health services, including mental health and school-based services	Intermittent iron and folic acid supplementation (menstruating women where anaemia is a public health problem)
Management of acute and chronic heart failure	Intermittent folic acid fortification for postpartum, non-anaemic pregnant women
Management of chronic kidney disease	Calcium supplementation for prevention and treatment of pre-eclampsia and eclampsia
Advanced care for acute myocardial infarction (i.e., beyond aspirin) and for acute peripheral vascular disease	Clean postnatal practices (regarded as a quality intervention in DCP3, not a specific health service)
Management of acute respiratory diseases	Chlorhexidine for cord care
More intensive efforts to screen for HIV (e.g., household- and community-based interventions)	Support for maternal depression
Pre-exposure prophylaxis for HIV	Management of mastitis
INH preventive therapy for select populations at risk of TB	Feeding counseling and support for low birth weight infants (implied in DCP3 nutrition recommendations, but not specified)
Active case finding for TB	Nurturing care counseling for early child development
Referral services for TB (specific mention of MDR-TB, XDR-TB)	Vitamin A for measles treatment in children
HBV and HCV treatment	Screening mammography
Malaria "elimination" interventions for countries with lower transmission	Colorectal cancer screening
Presumptive treatment of malaria in select settings	Post-cancer surveillance
Management of adult febrile illness (severe and non-severe)	Tobacco quit lines and mCessation
Rehabilitation and disability services	Physical activity brief advice as part of routine care
Screening and treatment of a variety of congenital and genetic disorders	HIV/AIDS community mobilisation
Management of musculoskeletal disorders	Post-exposure prophylaxis

More detailed and expanded palliative care services (as compared to WHO)	Collaborative TB/HIV activities, and management of comorbidities
	Active case finding for NTDs

Finally, while both studies report their estimates by World Bank income group, the country classifications drew on different World Development Indicators data (2014 for DCP3 and 2016 for WHO, respectively). Table A12 illustrates the differences in which countries were included in the LIC and lower-MIC categories and, in the case of the WHO analysis, the upper-MIC category. These differences account for much of the divergence in cost estimates.

Table A12. Countries included in the DCP3 and WHO analyses of UHC costs

**Low-income**		**Lower-middle-income**		**Upper-middle-income**	
**DCP3**	**WHO**	**DCP3**	**WHO**	**DCP3**	**WHO**
n = 34	*n = 27*	*n = 49*	*n = 22*	*n = 0*	*n = 18*
Afghanistan	Afghanistan	Armenia			Algeria
Bangladesh			Bangladesh		Angola
Benin	Benin	Bhutan			Azerbaijan
Burkina Faso	Burkina Faso	Bolivia			Brazil
Burundi	Burundi	Cabo Verde			China
Cambodia			Cambodia		Colombia
Cen. Afr. Republic	Cen. Afr. Republic	Cameroon	Cameroon		Dom. Republic
Chad	Chad	Congo			Ecuador
Comoros	Comoros	Cote d'Ivoire	Côte d'Ivoire		Iran
Dem. Rep. Korea		Djibouti			Iraq
Dem. Rep. Congo	Dem. Rep. Congo	Egypt	Egypt		Kazakhstan
Eritrea	Eritrea	El Salvador			Malaysia
Ethiopia	Ethiopia	Georgia			Mexico
Gambia	Gambia	Ghana	Ghana		Peru
Guinea	Guinea	Guatemala			Romania
Guinea-Bissau	Guinea-Bissau	Guyana			South Africa
Haiti	Haiti	Honduras			Thailand
Kenya		India	India		Turkey
Liberia	Liberia	Indonesia	Indonesia		
Madagascar	Madagascar		Kenya		
Malawi	Malawi	Kiribati			
Mali	Mali	Kyrgyzstan			
Mozambique	Mozambique	Laos			
Myanmar		Lesotho			
Nepal	Nepal	Mauritania			
Niger	Niger	Fed. St. Micronesia			
Rwanda	Rwanda	Mongolia			
Sierra Leone	Sierra Leone	Morocco	Morocco		
Somalia			Myanmar		
	South Sudan	Nicaragua			
Tajikistan		Nigeria	Nigeria		
Togo	Togo	Pakistan	Pakistan		
Uganda	Uganda	Papua New Guinea			
Tanzania	Tanzania	Paraguay			
Zimbabwe		Philippines	Philippines		
		Moldova			

		Samoa			
		Sao Tome/Principe			
		Senegal			
		Solomon Islands			
		South Sudan			
		Sri Lanka	Sri Lanka		
		Sudan	Sudan		
		Swaziland			
		Syria			
		Timor-Leste			
			Tunisia		
		Ukraine	Ukraine		
		Uzbekistan	Uzbekistan		
		Vanuatu			
		Viet Nam	Viet Nam		
		Palestine			
		Yemen	Yemen		
		Zambia			
			Zimbabwe		

Notes: countries in blue are included in the same income group and were included in both studies. Countries in red were included in different income groups but were included in both studies. Countries in green were only included in the DCP3 analysis. Countries in purple were only included in the WHO analysis. World Bank classifications were as of July 2014 for DCP3 and July 2016 for WHO.

The WHO costing study is arguably the most sophisticated and comprehensive to date, particularly with regard to estimating total health system investments required to achieve UHC (differences in the definition of "UHC" notwithstanding – as highlighted in the discussion section of the main manuscript). We view the DCP cost model as having complementary value to the WHO analysis, for several reasons. First, DCP3's focus was on priority setting for health, i.e., assessing efficient combinations of health interventions in order to achieve disease-specific and overall health targets. Our model looks at UHC through the lens of an intervention package where specific interventions can be readily added or deleted to rapidly assess the cost implications of changing the components of the HBP. Second, we acknowledge the tradeoff between analytic sophistication and ease of understanding model results. Our model errs on the side of the latter, recognising that more sophisticated approaches might produce more precise results and/or factor in country-specific contextual factors that we did not include. Third—and related to the previous two points—our online visualisation tool is designed to provide a high degree of transparency about our model inputs and assumptions and allow the user to change any inputs they do not agree with to get an alternative set of estimates.

References

1. Jamison DT, Alwan A, Mock CN, et al. Universal health coverage and intersectoral action for health: key messages from Disease Control Priorities, 3rd edition. *Lancet* 2018; **391**(10125): 1108-20.
2. Watkins DA, Jamison DT, Mills T, et al. Universal Health Coverage and Essential Packages of Care. In: rd, Jamison DT, Gelband H, et al., eds. Disease Control Priorities: Improving Health and Reducing Poverty. Washington (DC); 2017.
3. Horton S. Cost-Effectiveness Analysis in Disease Control Priorities, Third Edition. In: rd, Jamison DT, Gelband H, et al., eds. Disease Control Priorities: Improving Health and Reducing Poverty. Washington (DC); 2017.
4. Watkins DA, Nugent R, Saxenian H, et al. Intersectoral Policy Priorities for Health. In: rd, Jamison DT, Gelband H, et al., eds. Disease Control Priorities: Improving Health and Reducing Poverty. Washington (DC); 2017.
5. Johansson KA, Økland J-M, Skaftun EK, et al. Measuring Baseline Health with Individual Health-Adjusted Life Expectancy (iHALE). *medRxiv* 2019: 19003814.
6. Jamison DT, Summers LH, Alleyne G, et al. Global health 2035: a world converging within a generation. *Lancet* 2013; **382**(9908): 1898-955.
7. Nachbar J. Comparative statics. In: Durlauf SN, Blume LE, editors. The New Palgrave Dictionary of Economics Online. 2nd ed: Palgrave Macmillan; 2008.
8. Stenberg K, Hanssen O, Edejer TT, et al. Financing transformative health systems towards achievement of the health Sustainable Development Goals: a model for projected resource needs in 67 low-income and middle-income countries. *Lancet Glob Health* 2017; **5**(9): e875-e87.
9. WHO (World Health Organization). Global status report on noncommunicable diseases 2010. 2011. http://www.who.int/nmh/publications/ncd_report2010/en/ (accessed March 30, 2013.
10. UN. World Population Prospects: The 2017 Revision. New York: United Nations Department of Economic and Social Affairs, Population Division; 2017.
11. Darroch J, Singh S, Weissman E. Adding it up: The costs and benefits of investing in sexual and reproductive health 2014—estimation methodology. Appendix B: Estimating sexual and reproductive health program and systems costs.: Guttmacher Institute; 2016.
12. GBD 2015 Disease and Injury Incidence and Prevalence Collaborators. Global, regional, and national incidence, prevalence, and years lived with disability for 310 acute and chronic diseases and injuries, 1990-2015: a systematic analysis for the Global Burden of Disease Study 2015. *Lancet* 2016; **388**: 1545-602.
13. WHO. Global health observatory (GHO) data. 2016. http://www.who.int/gho/en/ (accessed 4 June 2016).
14. Seshadria SR, Jha P, Sati P, Gauvreau C, Ram U, Laxminarayan R. Karnataka's roadmap to improved health: cost eff ective solutions to address priority diseases, reduce poverty and increase economic growth. Bangalore: Azim Premji University, 2015.
15. High-Level Taskforce. Working Group 1 technical report. Constraints to scaling up and costs. High Level Taskforce (HLTF) on innovative international financing for health systems. Geneva: World Health Organization; 2009.
16. Bertram MY, Sweeny K, Lauer JA, et al. Investing in non-communicable diseases: an estimation of the return on investment for prevention and treatment services. *Lancet* 2018; **391**(10134): 2071-8.
17. Prinja S, Nandi A, Horton S, Levin C, Laxminarayan R. Costs, Effectiveness, and Cost-Effectiveness of Selected Surgical Procedures and Platforms. In: Debas HT, Donkor P, Gawande A, Jamison DT, Kruk ME, Mock CN, eds. Essential Surgery: Disease Control Priorities, Third Edition (Volume 1). Washington (DC); 2015.
18. Horton S, Levin C. Cost-Effectiveness of Interventions for Reproductive, Maternal, Neonatal, and Child Health. In: Black RE, Laxminarayan R, Temmerman M, Walker N, eds. Reproductive, Maternal, Newborn, and Child Health: Disease Control Priorities, Third Edition (Volume 2). Washington (DC); 2016.
19. Horton S, Gauvreau CL. Cancer in Low- and Middle-Income Countries: An Economic Overview. In: Gelband H, Jha P, Sankaranarayanan R, Horton S, eds. Cancer: Disease Control Priorities, Third Edition (Volume 3). Washington (DC); 2015.
20. Levin C, Chisholm D. Cost-Effectiveness and Affordability of Interventions, Policies, and Platforms for the Prevention and Treatment of Mental, Neurological, and Substance Use Disorders. In: Patel V, Chisholm D, Dua T, Laxminarayan R, Medina-Mora ME, eds. Mental, Neurological, and Substance Use Disorders: Disease Control Priorities, Third Edition (Volume 4). Washington (DC); 2016.
21. Gaziano TA, Suhrcke M, Brouwer E, Levin C, Nikolic I, Nugent R. Costs and Cost-Effectiveness of Interventions and Policies to Prevent and Treat Cardiovascular and Respiratory Diseases. In: rd, Prabhakaran D, Anand S, et al., eds. Cardiovascular, Respiratory, and Related Disorders. Washington (DC); 2017.

22. Watkins DA, Dabestani N, Nugent R, Levin C. Interventions to Prevent Injuries and Reduce Environmental and Occupational Hazards: A Review of Economic Evaluations from Low- and Middle-Income Countries. In: rd, Mock CN, Nugent R, Kobusingye O, Smith KR, eds. Injury Prevention and Environmental Health. Washington (DC); 2017.
23. Brouwer ED, Watkins D, Olson Z, Goett J, Nugent R, Levin C. Provider costs for prevention and treatment of cardiovascular and related conditions in low- and middle-income countries: a systematic review. *BMC Public Health* 2015; **15**: 1183.
24. Drummond MF, Sculpher MJ, Torrance GW, O'Brien BJ, Stoddart GL. Methods for the economic evaluation of health care programmes. 3rd ed. New York: Oxford University Press; 2005.
25. Parker M. Global inflation: the role of food, housing, and energy prices. ECB Working Paper 2024. 2017. https://www.ecb.europa.eu/pub/pdf/scpwps/ecbwp2024.en.pdf?ddefb6baab7a8afe961b7e02fc419d93.
26. Krakauer E, Kwete X, Verguet S, et al. Palliative care and pain control. 3 ed. Washington, D.C.: World Bank; 2018.
27. World Bank. People, pathogens, and our planet. Volume 2: The economics of One Health. Department of Agriculture and Rural Development. Washington, D.C.: World Bank 2012.
28. Fleming K, Naidoo M, Wilson M, et al. High quality diagnosis: an essential pathology pacakge. 3 ed. Washington, D.C.: World Bank; 2018.
29. Bautista-Arredondo S, Colchero MA, Amanze OO, et al. Explaining the heterogeneity in average costs per HIV/AIDS patient in Nigeria: The role of supply-side and service delivery characteristics. *PloS one* 2018; **13**(5): e0194305.
30. Stenberg K, Hanssen O, Bertram M, et al. Guide posts for investment in primary health care and projected resource needs in 67 low-income and middle-income countries: a modelling study. *Lancet Glob Health* 2019; **7**(11): e1500-e10.
31. Stenberg K, Axelson H, Sheehan P, et al. Advancing social and economic development by investing in women's and children's health: a new Global Investment Framework. *Lancet* 2014; **383**(9925): 1333-54.
32. Schwartlander B, Stover J, Hallett T, et al. Towards an improved investment approach for an effective response to HIV/AIDS. *Lancet* 2011; **377**(9782): 2031-41.
33. Reid MJA, Arinaminpathy N, Bloom A, et al. Building a tuberculosis-free world: The Lancet Commission on tuberculosis. *Lancet* 2019; **393**(10178): 1331-84.
34. Feachem RGA, Chen I, Akbari O, et al. Malaria eradication within a generation: ambitious, achievable, and necessary. *Lancet* 2019; **394**(10203): 1056-112.
